# One‐Stone‐Two‐Birds Carrier‐Free Nano‐Cocktail Enables Synergistic Eradication of Cancer Cells/Stem Cells in Breast Cancer Treatment

**DOI:** 10.1002/EXP.20240259

**Published:** 2025-12-22

**Authors:** Tongyao Zhao, Yao Chen, Haimeng Yuan, Shuqian Yang, Hongyuan Zhang, Yuequan Wang, Shenwu Zhang, Qin Chen, Jin Sun, Zhonggui He, Cong Luo

**Affiliations:** ^1^ Department of Pharmaceutics Wuya College of Innovation Shenyang Pharmaceutical University Shenyang P. R. China; ^2^ School of Electrical and Electronic Engineering Nanyang Technological University (NTU) Singapore Singapore; ^3^ Department of Pharmacy Cancer Hospital of China Medical University Liaoning Cancer Hospital and Institute Shenyang P. R. China; ^4^ Joint International Research Laboratory of Intelligent Drug Delivery Systems Ministry of Education Shenyang Pharmaceutical University Shenyang P. R. China

**Keywords:** cancer stem cells, docetaxel prodrug, nano‐cocktail, salinomycin, synergistic chemotherapy

## Abstract

Cancer stem cells (CSCs) are widely recognized as the culprits of chemoresistance, tumor metastasis, and relapse. Moreover, most chemotherapeutic drugs not only fail to eliminate CSCs effectively, but also induce the acquisition of stemness characteristics in non‐stem cancer cells. Herein, we propose a cancer cells/CSCs double‐killing modality for breast cancer treatment. Specifically, a carrier‐free nano‐cocktail is developed through the precise co‐assembly of a redox‐responsive docetaxel (DTX) dimeric prodrug and salinomycin (SAL, an anti‐CSCs drug). Precision combination of DTX and SAL not only shows synergistic tumor‐killing activity, but also sharply reduces the proportion of CSCs in tumors. More importantly, tumor‐specific prodrug activation‐initiated drug release from the nano‐cocktail confers high drug co‐delivery efficiency and low off‐target toxicity risk. As expected, such a one‐stone‐two‐birds nanomedicine presents satisfactory performance on tumor stemness eradication, antitumor responses, and safety in both xenograft and orthotopic 4T1 breast cancer mouse models. This study advances cancer cells/CSCs double‐killing nanotherapeutics towards clinical breast cancer therapy.

## Graphical abstract

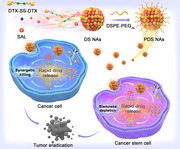

## 1 Introduction

Cancer remains a serious threat to global public health [1, 2, 3]. Despite many therapeutic targets and anticancer drugs discovered and developed, clinical anticancer outcomes are still far from satisfactory [4, 5, 6, 7, 8]. The reasons for such a situation are rather complicated. Among them, the existence of cancer stem cells (CSCs) has been recognized as a primary factor contributing to therapeutic resistance and tumor recurrence [9]. Accounting for only small populations of tumor tissues, CSCs with unique self‐renewal capability and multi‐differentiation potential have been identified as the ringleaders of drug resistance and tumor recurrence [10]. The discovery of CSCs was reported in acute myeloid leukemia (AML) for the first time [11]. Subsequently, considerable CSCs were widely found not only in non‐solid tumors but also in multifarious solid tumors, including breast cancer, hepatic carcinoma, and glioblastoma [12, 13, 14]. Particularly, breast cancer, as the most frequent cancer type and the primary cause of cancer‐related mortality among women globally, continues to pose significant challenges in clinical treatment [15]. At present, chemotherapy remains one of the principal clinical therapeutic approaches available for breast cancer management [16]. However, despite the potent killing effect on the bulky breast cancer cells, CSCs colonized in breast tumor tissues are resistant to most chemotherapeutic drugs [17]. Not only that, chemotherapeutic drugs such as docetaxel (DTX) have been found to trigger the phenotypic transformation from non‐stem cancer cells to CSCs, dramatically aggravating chemotherapy resistance and even leading to treatment failure [18, 19, 20].

Given the important role in tumorigenesis and progression, CSCs have received wide attention as an attractive target for cancer treatment [21, 22]. So far, multiple anti‐CSCs strategies have been developed, including differentiation induction and destruction approaches [23]. Numerous differentiation‐inducing agents, including all‐trans retinoic acid (ATRA), have been reported to efficiently reduce stemness by inducing differentiation of CSCs [24]. However, many practical difficulties exist in inducing stem cell differentiation in vivo, since CSCs are usually surrounded by bulky cancer cells and immune cells, making it difficult for differentiation‐inducing drugs to easily reach the CSCs niches. In addition to differentiation induction strategy, certain drugs have been identified as potent agents against CSCs. Among them, Weinberg et al. found and reported the potent anti‐CSCs activity of salinomycin (SAL) in 2009 [25]. Before that, SAL found commercial application in poultry farming as an antibacterial and anticoccidial agent [26]. SAL effectively eliminates CSCs by intervention of CSCs‐associated cellular signaling pathways (e.g., Wnt and MAPK pathways), sequestration of iron in lysosomes, induction of DNA damage and endoplasmic reticulum (ER) stress, and suppression of marker expression on CSCs [26, 27]. Clinical case results revealed that both SAL‐involved monotherapy and combination regimens exerted advanced cancer regression [26]. However, despite potent anti‐CSCs activity, the undesirable properties of SAL still greatly hinder its clinical application, such as low water solubility [28], poor pharmacokinetics, and off‐target differentiation inhibition on normal mesenchymal stem cells [23, 29]. Moreover, SAL showed much weaker cytotoxicity against normal cancer cells than most chemotherapeutic drugs, which also makes it difficult to reach the CSCs niches surrounded by bulky cancer cells and immune cells in solid tumors [30, 31]. Given all this, it is necessary to devise chemotherapy/anti‐CSCs combination regimens to address the twin challenges of inadequate antitumor effects of anti‐CSCs monotherapy and chemotherapy‐induced tumor stemness upregulation.

Feasible drug combination modalities depend heavily on favorable drug co‐delivery performance, satisfactory synergistic effect, and manageable off‐target toxicity risks [32, 33, 34]. Over decades, biomedical nanotechnology has greatly changed drug delivery patterns by improving undesirable properties of drugs [2, 8, 35, 36], overcoming delivery barriers, and endowing drugs with targeting and smart delivery characteristics [6, 35]. Significantly, nanomedicines not only enrich drug delivery modalities for a single drug [37, 38], but also provide versatile co‐delivery nanoplatforms for multimodal cancer therapy [32, 39]. However, despite the huge potential of nanotherapeutics, carrier material‐based co‐delivery nanomedicines face certain challenges in drug co‐loading and co‐delivery in vivo [40, 41]. Particularly, owing to the fact that there is a wide difference in affinity between different drugs and carrier materials, co‐encapsulation of two or more drugs in traditional nanocarriers usually suffers from poor co‐loading performance, inconvenient dose ratio modulation, premature drug leakage, desynchrony in co‐delivery, and drug release in the body [42]. Additionally, an expanding body of evidence suggests that clinical translation of carrier material‐based nanomedicines is significantly hindered by the pharmaceutical excipient availability of organic or inorganic carrier materials [43]. In recent times, carrier‐free nanomedicines formed through the co‐assembly of small molecules represent a prospective co‐delivery modality, offering unique benefits in terms of simple fabrication processes [32], superior drug co‐loading efficiency [8], and synchronous co‐delivery features [44]. Based on this distinct nanofabrication technology, we proposed that developing a precisely hybrid nanomedicine co‐assembled by a tumor stimuli‐activatable chemotherapeutic prodrug and an anti‐CSCs drug would not only achieve a cancer cells/CSCs double‐killing effect, but also significantly reduce toxicity risks of drug combinations by virtue of tumor‐specific prodrug activation and anti‐CSCs drug release.

**FIGURE 1:**
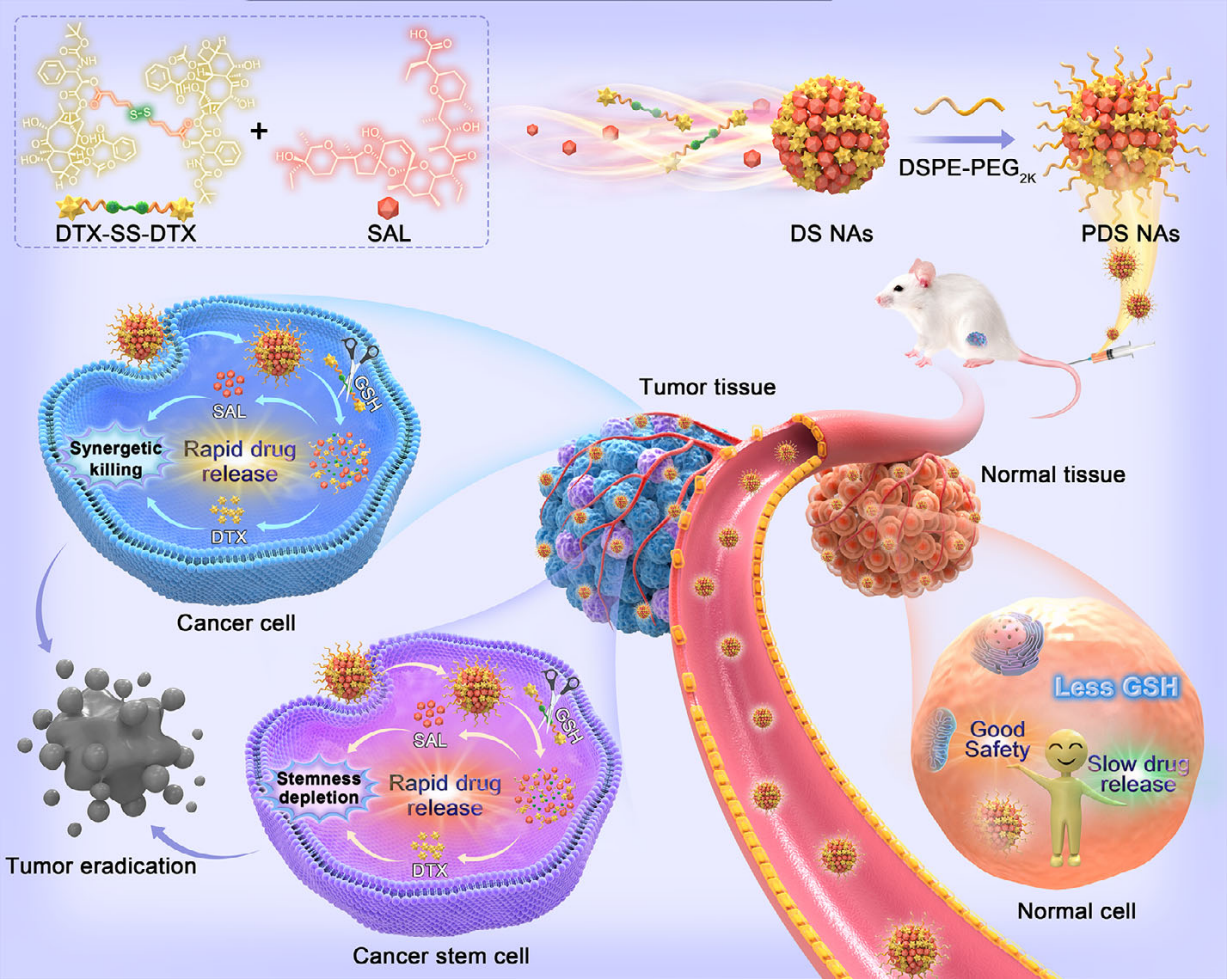
Schematic illustration of molecularly engineered one‐stone‐two‐birds nano‐cocktail for cancer cells/CSCs double‐killing. The nano‐cocktail was precisely fabricated by a disulfide‐bridged docetaxel (DTX) dimeric prodrug (DTX‐SS‐DTX, DSSD) and the potent anti‐CSCs agent salinomycin (SAL) based on the molecular nanoassembly technique. Following PEGylation modification, such a nano‐cocktail enabled on‐demand prodrug activation and site‐specific SAL release in response to GSH overproduction in cancer cells and CSCs rather than in normal cells with less GSH, thereby realizing the synergistic eradication of cancer cells/CSCs in an efficient and safe manner.

To test our hypothesis, a nano‐cocktail was fabricated through precise nanoassembly of a redox‐responsive dimeric prodrug of docetaxel (DTX‐SS‐DTX, DSSD) and SAL (Figure 1). DSSD was facilely synthesized by conjugating two DTX molecules via the disulfide bond as a redox‐responsive linkage, which can be rapidly activated by the high intracellular glutathione (GSH) concentrations in both tumor cells and CSCs [45]. We observed that DSSD can readily co‐assemble with SAL to form uniform nanoassemblies (NAs) across broad molar ratios from 5:1 to 1:5, while its parent drug DTX exhibited poor nanoassembly performance with SAL under the same conditions. Electrostatic and hydrophobic forces were revealed to be the key drivers for the co‐assembly process of DSSD and SAL. The formulations were optimized by comprehensively assessing the co‐assembly performance of NAs and the cooperativity index (CI) of DSSD and SAL against 4T1 breast cancer cells. To enhance colloidal stability and pharmacokinetic profiles, a minor amount of polyethylene glycol (PEG) polymer (DSPE‐PEG_2K_) was decorated on DSSD/SAL NAs. The PEGylated nano‐cocktail showed high DTX/SAL co‐loading efficacy, favorable colloidal stability, prolonged systemic circulation, efficient site‐specific accumulation in tumors, and prodrug activation‐gated on‐demand release patterns of DTX and SAL. Notably, SAL specifically enhanced the cytotoxic effects of DTX towards cancer cells and CSCs but not that of normal cells, displaying a selective chemotherapy enhancement feature. As expected, such a one‐stone‐two‐birds nano‐cocktail exerted efficient tumor stemness eradication and synergistic tumor‐killing effects in both xenograft and orthotopic 4T1 breast cancer mouse models. More importantly, it showed good safety owing to stimulus‐triggered prodrug activation and site‐specific drug release from the nano‐cocktail. As far as we know, this represents the inaugural effort in developing a carrier‐free nano‐cocktail on the basis of molecular nanoassembly of tumor stimuli‐activatable chemotherapeutic prodrugs and anti‐CSCs agents. This study provides a novel cancer cells/CSCs double‐killing paradigm with high efficiency and safety for synergistic chemotherapy.

## 2 Results

### 2.1 Synthesis of a Dimeric Prodrug of DTX

This project commenced with an attempt to address the dual challenges of chemotherapy‐induced tumor stemness evolution and CSCs‐mediated chemoresistance. In this case, the combination of chemotherapeutic drugs with anti‐CSCs agents seemed to be a feasible way out of this clinical dilemma. Nevertheless, the toxicity risks would sharply increase in the case of intravenous administration of two or more drugs in one dose, especially with the involvement of cytotoxic chemotherapy drugs. Over decades, the prodrug strategy has been extensively utilized to enhance delivery efficiency and reduce drug toxicity [37]. Particularly, tumor‐specific activation of prodrugs can be achieved through inserting certain chemical linkages, such as disulfide and thioether bonds, into the prodrug structure [45, 46, 47]. In the present study, we first synthesized a dimeric prodrug (DTX‐SS‐DTX) by conjugating two DTX molecules via a disulfide bond according to the previously reported method (Figure S1) [48], abbreviated as DSSD. Mass spectrometry (MS) and nuclear magnetic resonance (NMR) spectroscopy, which includes both ^1^H NMR and ^13^C NMR, confirmed the successful synthesis of DSSD (Figure S2).

### 2.2 Nanoassembly Performance and Synergistic Effect of DSSD and SAL

As previously mentioned, we aimed to construct a hybrid nanoassembly of chemotherapy drug/anti‐CSCs agent to solve the problems of chemotherapy‐induced tumor stemness evolution and CSCs‐mediated chemoresistance. In preliminary attempts, we observed that neither DSSD nor SAL was capable of forming uniform nanoassemblies (NAs) through self‐assembly via a simple one‐step nanoprecipitation technique. Interestingly, DSSD was readily able to co‐assemble with SAL to form NAs under identical conditions without relying on any additional excipients. Furthermore, it was worth noting that the molar ratios of DSSD and SAL in NAs enabled precise adjustment over a broad range from 5:1 to 1:5, demonstrating remarkable co‐assembly capability (Table S1). Significantly, we further found that SAL, as an excellent anti‐CSCs agent, exhibited a synergistic antitumor effect with DSSD. As presented in Table S2, DSSD and SAL showed excellent synergy effects at molar ratios of 5:1 to 1:5, as evidenced by cooperativity index (CI) values consistently below 1. The most prominent synergistic cytotoxicity of DSSD and SAL was observed when the molar proportion was 1:1, with the smallest CI value of 0.294 (Table S2). Based on these findings, the optimal DSSD/SAL molar ratio of 1:1 was selected to fabricate dual‐drug NAs for subsequent experimental studies.

### 2.3 Elaborate Fabrication of a Molecularly Assembled Nano‐Cocktail

**FIGURE 2:**
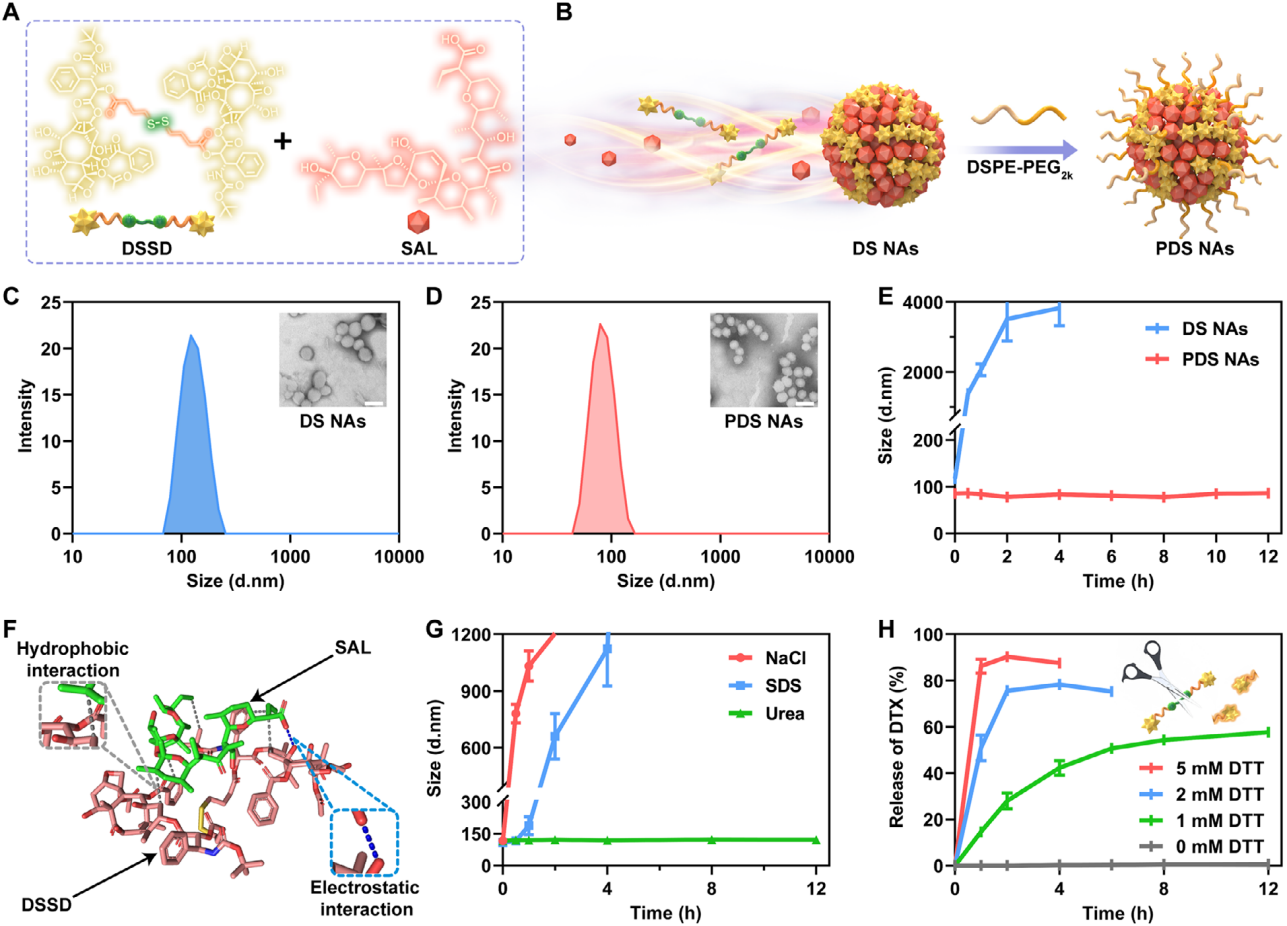
Preparation and characterization of a carrier‐free nano‐cocktail. (A) The chemical structures of DSSD and SAL. (B) Diagrammatic illustration of the procedure for the co‐assembly of DSSD/SAL NAs. (C,D) Size distribution and TEM images of DS NAs and PDS NAs. Scale bar represents 100 nm. (E) Stability evaluation of DS NAs and PDS NAs in PBS (pH 7.4) (*n* = 3). (F) Molecular docking simulation illustrating the molecular interactions involved in the co‐assembly of DSSD and SAL. (G) Particle size variation of DS NAs after incubation with NaCl, SDS, and urea (*n* = 3). (H) Release profiles of DTX from PDS NAs with varying DTT concentrations in release medium (*n* = 3). Data represented as mean ± SD.

The bare DSSD/SAL NAs (named DS NAs) without any surface modification were fabricated by the single‐step nanoprecipitation process with the molar ratio of 1:1 (Figure 2A,B). To enhance the colloidal stability and pharmacokinetic profiles, a minor proportion of DSPE‐PEG_2K_ was employed as a PEGylation modifier to fabricate PEGylated co‐assemblies. For the purpose of optimizing the ratio of DSPE‐PEG_2K_, varying amounts of DSPE‐PEG_2K_ were used in the preparation of PEGylated DSSD/SAL NAs. Based on comprehensive evaluations involving particle diameter, zeta potential, and polydispersity index (PDI), we found that 20 wt% DSPE‐PEG_2K_ was optimal for fabricating the PEGylated DSSD/SAL NAs, designated as PDS NAs (Figure 2A,B; Table S3). It is crucial to note that the PDS NAs exhibited remarkable drug‐loading efficiency, achieving 50% for DTX and 23% for SAL (Table S4). The particle sizes of DS NAs and PDS NAs were ≈121 and 82 nm, corresponding to the negative *zeta* potentials of ≈−25 and −30 mV, respectively (Figure 2C,D; Figure S3). Notably, the particle size of DSSD/SAL NAs exhibited a significant reduction following modification with DSPE‐PEG_2K_, suggesting the stabilizing effect of PEGylation on DSSD/SAL NAs with a more compact nanostructure (Figure 2C,D). Moreover, both DS NAs and PDS NAs exhibited a regular spherical morphology according to the images of transmission electron microscopy (TEM) shown in Figure 2C,D.

We proceeded to examine the stability of DS NAs and PDS NAs through incubation in phosphate‐buffered saline (PBS, pH 7.4) at 37°C. The particle size of PDS NAs remained almost constant throughout the incubation period, whereas DS NAs displayed poor colloidal stability with a sharp particle size increase under identical experimental conditions, as illustrated in Figure 2E. Moreover, PDS NAs also showed excellent stability when stored in PBS (pH 7.4) containing 10% serum over 12 h (Figure S4) and at a temperature of 4°C for one month (Figure S5). Additionally, PDS NAs could be readily lyophilized for long‐term storage. As shown in Figure S6, the lyophilized PDS NAs displayed favorable stability and dispersibility upon reconstitution with minimal changes in particle size and retention of their spherical morphology. These results suggested that PEGylation modification endowed PDS NAs with excellent stability, which would in turn be advantageous for extending the systemic circulation duration of NAs. As such, we successfully fabricated a nano‐cocktail based on the molecular prodrug/drug nanoassembly modality, which was expected to effectively respond to the dilemma of tumor stemness and chemoresistance through simultaneously killing cancer cells and CSCs.

### 2.4 Insight Into the Co‐Assembly Mechanism of Nano‐Cocktail

Furthermore, we conducted preliminary investigations into the mechanisms involved in co‐assembly between DSSD and SAL. Molecular docking simulation methods were utilized for the clarification of the driving forces underlying the nanoassembly process. As illustrated in Figure 2F, electrostatic interactions and hydrophobic forces were determined to be the predominant intermolecular interactions driving the formation of DSSD/SAL nanoassembly. To further verify this, three frequently used intermolecular force disruptive agents (sodium chloride [NaCl], sodium dodecyl sulfate [SDS], and urea) were added to the hybrid NAs to destroy the electrostatic interactions, hydrophobic effects, and hydrogen bonds between DSSD and SAL, respectively. As illustrated in Figure 2G, the nanostructure of DS NAs remained stable even in the presence of urea, which revealed that hydrogen‐bonding interactions had a negligible impact on NAs formation. In stark contrast, the particle size of DS NAs increased conspicuously within a short period after incubation with NaCl and SDS. These findings aligned with the molecular docking simulation results, further substantiating the crucial roles of electrostatic interactions and hydrophobic forces in the co‐assembly process.

### 2.5 Reduction‐Responsive Prodrug Activation and Nanoassembly Collapse

Successful nanoassembly and favorable performance of the nano‐cocktail prompted us to further investigate its reduction‐responsive prodrug activation and nanoassembly collapse. First, release media containing serial concentrations of dithiothreitol (DTT, a prevalent analogue of GSH) were employed for the evaluation of in vitro release profiles of DTX. As illustrated in Figure 2H, PDS NAs showed concentration‐dependent DTX release behaviors in the presence of DTT. Remarkably, approximately 90% of DTX was released within 2 h triggered by DTT (5 mM). In contrast, there is very little DTX released from PDS NAs in the absence of DTT. Reduction‐responsive prodrug activation and DTX release from the nano‐cocktail would certainly benefit from reducing or even avoiding premature drug leakage and off‐target toxicity associated with the DTX/SAL combination. Moreover, we also observed a sharp increase in the particle size of PDS NAs along with nanoassembly collapse triggered by DTT (Figure S7). Regrettably, owing to the serious interference of DSSD with the quantitative method of SAL, we failed to accurately assess the release characteristics of SAL from the nano‐cocktail. Still, tumor‐specific nanoassembly collapse caused by reduction‐responsive prodrug activation would certainly trigger rapid SAL release from the nano‐cocktail. Given the high GSH levels in both tumor cells and CSCs, the nano‐cocktail was expected to achieve site‐specific prodrug activation‐initiated drug release at tumor sites, thereby realizing cancer cells/CSCs double‐killing in an efficient and safe manner.

### 2.6 Cellular Uptake

**FIGURE 3:**
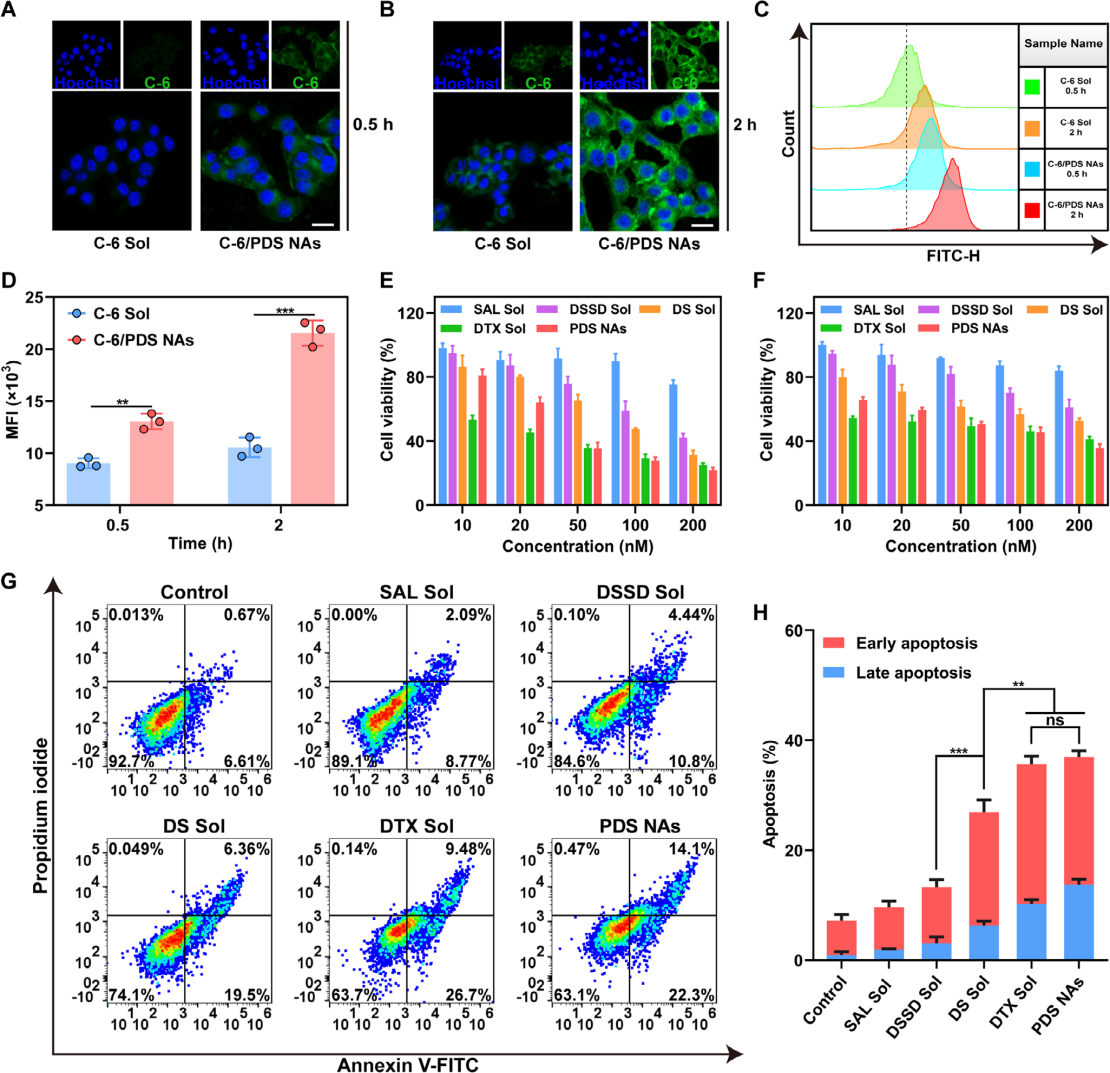
Cellular uptake and synergistic cytotoxicity of nano‐cocktail. (A,B) Cellular uptake images of C‐6 and C‐6/PDS NAs in 4T1 cancer cells. Scale bar represents 10 µm. (C) Evaluation of cellular uptake using flow cytometry in 4T1 cancer cells and (D) quantification at 0.5 and 2 h (*n* = 3). (E) The cytotoxicity on 4T1 cancer cells following various treatments (*n* = 3). (F) Cytotoxic effects of different formulations against MCF‐7 cancer cells (*n* = 3). (G) Apoptosis analysis in 4T1 cancer cells after various treatments. (H) Quantitative assessment of apoptotic cells in 4T1 cancer cells (*n* = 3). Data are given as mean ± SD. p values: **p < 0.01, ***p < 0.001, ns means no significance.

Cellular internalization is of great significance to antitumor potency of nanomedicines. We next evaluated the cellular uptake behavior of nano‐cocktail with the aid of confocal laser scanning microscopy (CLSM). Coumarin‐6 (C‐6)‐labeled NAs (named C‐6/PDS NAs) were prepared to probe the cellular uptake capacity. The fluorescence intensity showed a time‐dependent increase in 4T1 cancer cells following treatment with both C‐6 Sol and C‐6/PDS NAs from 0.5 to 2 h (Figure 3A,B). Significantly, at equivalent concentrations of C‐6, the cellular fluorescent signals of C‐6/PDS NAs consistently exhibited stronger intensity than those of C‐6 Sol at both 0.5 and 2 h. Moreover, the quantitative data derived from flow cytometry provided further confirmation of the superior cellular uptake efficiency of C‐6/PDS NAs compared to C‐6 Sol under the same conditions (Figure 3C,D). High cellular uptake efficiency of NAs should be ascribed to specific endocytosis‐mediated internalization mechanisms of nanoparticles [38, 49]. These findings indicated that the nano‐cocktail demonstrated higher cellular uptake efficacy in comparison with the free drug solution.

### 2.7 Synergistic Cytotoxicity In Vitro

Encouraged by the outstanding colloidal stability, prodrug activation, and cellular uptake features of the nano‐cocktail, we further explored its synergistic cytotoxicity using MTT assays. We first evaluated cell viability after treatment with various free drugs and PDS NAs in 4T1 and MCF‐7 cancer cells, which are derived from different sources of breast cancer. As illustrated in Figure 3E,F, despite the weak antiproliferative effect of SAL, drug combination groups (DS Sol and PDS NAs) exhibited stronger cytotoxicity against both 4T1 and MCF‐7 cancer cells when compared to DSSD. This was consistent with the previous data (Table S2), further confirming the synergy effect of DSSD and SAL. Notably, the DSSD/SAL mixture (DS Sol) showed inferior cytotoxicity when compared to DTX Sol, which should be a consequence of the delayed release of active DTX from DSSD. By contrast, PDS NAs not only demonstrated much stronger cytotoxicity than that of DS Sol, but even slightly better than the parent drug DTX at high drug concentrations (Figure 3E,F). The in vitro antitumor superiority of PDS NAs over DS Sol can be ascribed to more effective and synchronous cellular uptake of DSSD and SAL after formulating them into a carrier‐free hybrid nanomedicine. More importantly, the comparable cytotoxicity of PDS NAs with DTX Sol suggested that rational design of a redox‐responsive dimeric prodrug and co‐assembly with SAL did not significantly reduce antineoplastic activity on the basis of safety. Following the various treatments, apoptosis was detected in 4T1 cancer cells using the Annexin V‐FITC and PI dual‐staining method. As illustrated in Figure 3G,H, PDS NAs elicited extensive tumor cell apoptosis, which was in agreement with the findings obtained from the MTT test (Figure 3E).

Furthermore, we expected that the wide disparity in redox stimuli between tumor cells and normal cells would result in minimal off‐target toxicity risk to the nano‐cocktail. Human hepatocytes (L02) and mouse fibroblast (3T3) cells were utilized for further investigation of cytotoxicity toward normal cells. As shown in Figures S8–S10, DTX Sol still showed potent killing effects against two kinds of normal cells. In contrast, all the DSSD‐containing formulations (DSSD Sol, DS Sol, and PDS NAs) exhibited negligible cytotoxicity against both L02 cells and 3T3 cells under identical conditions (Figures S8–S10). These outcomes well validated the feasibility and superiority of dimeric prodrug design in increasing tumor‐specific antineoplastic effects and decreasing off‐target toxicity.

### 2.8 Anti‐CSCs Efficacy In Vitro

As previously mentioned, our objective was to develop a nano‐cocktail with a dual‐killing mechanism capable of effectively eradicating both cancer cells and CSCs, thus providing a comprehensive coping strategy for chemotherapy‐induced tumor stemness evolution and CSCs‐mediated chemoresistance. The above data provided compelling evidence supporting the synergistic enhancement of tumor‐specific cytotoxicity by incorporating SAL into the nano‐cocktail. To further validate our design, we prioritized investigating the impact of SAL against breast cancer stem cells (BCSCs) in 4T1 cancer cells. Following SAL treatment, a notable decrease in the proportion of CD133^+^ CSCs and CD44^+^CD24^−^ CSCs was observed in 4T1 cancer cells compared to the untreated group, suggesting that SAL exhibited pronounced efficacy in eliminating CSCs (Figure S11).

**FIGURE 4:**
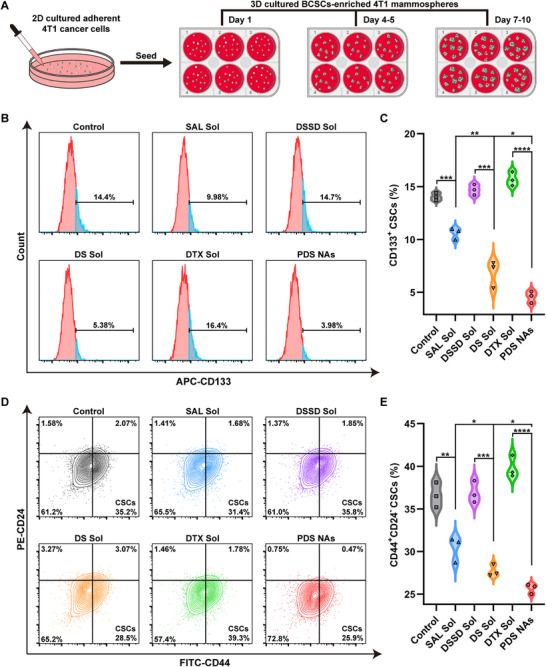
In vitro anti‐CSCs efficacy of the nano‐cocktail. (A) The process of enriching BCSC spheres derived from 4T1 cancer cells. (B) Representative flow cytometry images of CD133^+^‐identified CSCs following different treatments. (C) The proportion of CD133^+^ CSCs in 4T1 mammospheres exposed to different formulations (*n* = 3). (D) Representative images of flow cytometry analysis for the CD44^+^CD24^−^‐identified CSCs. (E) Quantification of CD44^+^CD24^−^ CSCs after various treatments. (*n* = 3). *p* values: *p < 0.05, **p < 0.01, ***p < 0.001, ****p < 0.0001.

On the basis of these findings, we promptly explored the anti‐CSCs potential of the nano‐cocktail (PDS NAs). Specifically, a CSC‐enriched 3D mammosphere model was established from 4T1 cancer cells using the serum‐free suspension culture method, as shown in Figure 4A. Afterward, the characteristic surface markers CD133^+^ and CD44^+^CD24^−^ were used to identify BCSCs in 3D‐cultured 4T1 mammospheres. According to the flow cytometry results (Figure S12), it was observed that the proportion of CD133^+^ and CD44^+^CD24^−^ CSCs in 3D mammospheres accounted for 12.08 ± 0.67% and 35.90 ± 1.80%, respectively, which were much higher than those derived from 2D cultured adherent 4T1 cancer cells (3.57 ± 0.27% and 5.44 ± 0.21%, respectively). This revealed the successful enrichment of prototypical stem‐like cells and established a foundation for further experimental investigations. A growing body of research suggested that CSCs demonstrate an overexpression in GSH levels, which conferred resistance to multiple therapeutic strategies [31]. Prior to investigating the anti‐CSCs effect, we assessed the GSH levels in both tumor cells and CSCs. Higher GSH levels were observed in CSCs compared to tumor cells (Figure S13), which would undoubtedly contribute to the high‐efficiency prodrug activation and SAL release of nano‐cocktail in CSCs.

Subsequently, flow cytometry analysis was carried out to evaluate the ratio of CD133^+^ CSCs and CD44^+^CD24^−^ CSCs in 3D mammospheres following treatment with various formulations. As expected, all the SAL‐containing formulations (SAL Sol, DS Sol, and PDS NAs) obviously reduced the ratio of BCSCs (Figure 4B–E). It was mentioned that the combined treatment groups (DS Sol and PDS NAs) exhibited a lower percentage of CSCs compared to mono‐treatment with SAL Sol, highlighting that SAL not only specifically enhanced the cytotoxicity of DTX against tumor cells but also possessed a significant advantage in synergistically killing CSCs. Moreover, the nano‐cocktail (PDS NAs) exhibited the best in vitro anti‐CSCs efficacy among all groups, which can be ascribed to its effective cellular internalization and DTX/SAL co‐release capacity. Notably, DTX Sol did not lead to a reduction in the population of BCSCs, instead, it induced an increased proportion of CD133^+^ CSCs and CD44^+^CD24^−^ CSCs in contrast to the untreated control group (Figure 4B–E). These findings indicated that DTX not only failed to eliminate BCSCs but also triggered the stemness evolution of tumor cells. By comparison, DSSD Sol did not cause similar phenotypic plasticity, which indicated that the prodrug strategy, to a certain degree, contributed to the relief of chemotherapy‐induced tumor stemness evolution. Overall, these results substantiated our initial hypothesis that such a nano‐cocktail had the potential to address the dual challenges of chemotherapy‐induced tumor stemness evolution and CSCs‐mediated chemoresistance.

### 2.9 Pharmacokinetics

**FIGURE 5:**
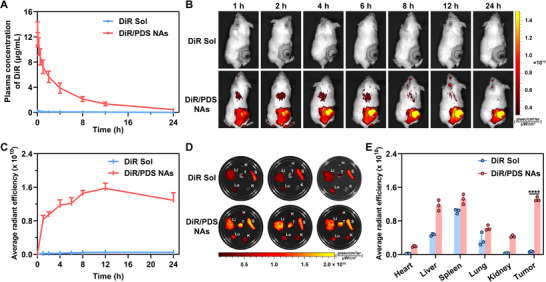
The pharmacokinetic and biodistribution of nano‐cocktail. (A) The pharmacokinetic profiles of DiR Sol and DiR/PDS NAs following intravenous injection in SD rats (*n* = 6). (B) Living images of subcutaneous 4T1 tumor‐bearing mice after DiR Sol and DiR/PDS NAs treatments. (C) Average radiant efficiency of tumor site at various time points (*n* = 3). (D) Ex vivo fluorescence imaging of main organs and tumors from mice treated with DiR Sol and DiR/PDS NAs at 24 h post‐injection. (E) Semi‐quantitative fluorescent analysis in major organs and tumors post‐injection at 24 h (*n* = 3). Data are presented as mean ± SD. p values: ****p < 0.0001.

The in vivo disposition fate certainly exerts a profound impact on the drug delivery and ultimate therapeutic efficacy of NAs. As previously discussed, PEGylation decoration was expected to stabilize the nano‐cocktail and prolong its blood circulation time in vivo. In this section, our attention was directed toward the pharmacokinetics of PDS NAs. Given that it is difficult to establish an appropriate analytical method for the simultaneous determination of DSSD, DTX, and SAL, a near‐infrared (NIR) fluorescent dye (DiR) was utilized to label the nano‐cocktail, defined as DiR/PDS NAs. We next assessed the pharmacokinetic characteristics of DiR Sol and DiR/PDS NAs by monitoring the plasma fluorescence intensity changes in Sprague–Dawley (SD) rats post intravenous injections. The primary pharmacokinetic metrics were computed and presented in Table S5. Following the intravenous administration of DiR Sol, the fluorescence signal exhibited rapid clearance as illustrated in Figure 5A. Conversely, DiR/PDS NAs with a larger area under the concentration‐time profile (AUC_0–24 h_) exhibited distinct superiority in prolonging blood circulation time over DiR Sol (Figure 5A; Table S5), which should be attributed to the favorable colloidal stability endowed by PEGylation modification. These results provided supportive evidence that the PEGylated nano‐cocktail markedly prolonged the duration of systemic circulation in vivo, which would undoubtedly be beneficial for the selective tumor accumulation of NAs through the enhanced permeability and retention (EPR) effect [31].

### 2.10 Biodistribution

Subsequently, we continued our investigation to study the biodistribution features of the nano‐cocktail in mice bearing subcutaneous 4T1 tumors. Similarly, DiR was applied to fabricate fluorescently‐labeled nano‐cocktail (DiR/PDS NAs). The intensity of DiR fluorescence in tumor tissues was monitored at predetermined time intervals using an in vivo imaging system (IVIS). As depicted in Figure 5B,C, DiR/PDS NAs exhibited an obviously enhanced fluorescence signal at tumor sites within 24 h than that of DiR Sol. Moreover, the peak accumulation in tumors was detected at 12 h. Such results could be attributed to the distinct advantages demonstrated by PDS NAs in terms of colloidal stability (Figure 2E) and pharmacokinetic behavior (Figure 5A). 24 h after injection, the mice were euthanized, and their main organs, along with tumor tissues, were collected for fluorescent imaging and semi‐quantitative analysis. As illustrated in Figure 5D,E, high tumor accumulation of DiR was observed in the mice receiving DiR/PDS NAs, aligning well with the results derived from in vivo imaging analysis.

### 2.11 Synergistic Antitumor Activity

**FIGURE 6:**
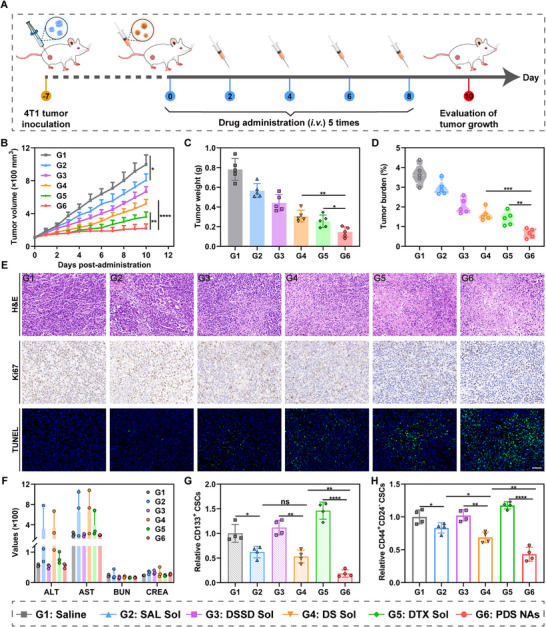
In vivo antitumor activity of the nano‐cocktail in mice bearing 4T1 xenograft tumors. (A) Diagram of the establishment and treatment scheme. (B) Tumor growth curves following different treatments (*n* = 5). (C) Tumor weight at the completion of treatment (*n* = 5). (D) Tumor burden of mice following various treatments (*n* = 5). (E) Images of H&E, Ki67, and TUNEL staining on tumor slices following different therapies. Scale bar represents 50 µm. (F) Parameters of the hepatorenal function following the last treatment (*n* = 3). (G) Proportion of CSCs detected using the CD133 surface marker (*n* = 4). (H) Proportion of CSCs identified by CD44 and CD24 surface markers (*n* = 4). Data represented as mean ± SD. *p* values: *p < 0.05, **p < 0.01, ***p < 0.001, ****p < 0.0001, ns means no significance.

Motivated by the favorable drug delivery performance and effective tumor accumulation capacity of the nano‐cocktail, we conducted further investigations to explore its synergistic antitumor activity in mice bearing 4T1 xenograft tumors. Once the tumors grew to around 100 mm^3^, we randomized the mice into six groups (five mice per group) and respectively treated them with saline, SAL Sol, DSSD Sol, DS Sol, DTX Sol, and PDS NAs. The experimental protocol was illustrated in Figure 6A, wherein various formulations were given intravenously to 4T1 tumor‐bearing mice on alternate days, totaling five injections, with each dose corresponding to 5 mg/kg of DTX. Additionally, the body weight and tumor size of each mouse were monitored and documented on a daily basis throughout the entire treatment process.

As illustrated in Figure 6B–D and Figures S14–S16, monotherapy with SAL and DSSD exhibited inferior antitumor efficacy, which might be attributed to their inefficient cytotoxicity and inadequate tumor accumulation. Moreover, the DSSD/SAL mixture (DS Sol) demonstrated enhanced antitumor efficacy relative to that of SAL Sol and DSSD Sol, indicating the synergistic antitumor potential of the DSSD/SAL combination. As expected, the nano‐cocktail (PDS NAs) exhibited the most pronounced inhibitory impact on tumor growth among all groups throughout the entire treatment period, with the highest tumor inhibitory rate (Figure S16) and lowest tumor burden (Figure 6D). Besides, the PDS NAs group demonstrated a significantly increased ratio of necrosis, reduced cell proliferation indicated by Ki67‐positive cells, and elevated degrees of apoptosis in TUNEL assays compared to the other groups (Figure 6E). Notably, DTX Sol also displayed potent antitumor activity at the same dose, while it was accompanied by a sharp body weight loss (Figure S17). Furthermore, the mice treated with SAL Sol and DS Sol also caused body weight reduction, hepatotoxicity, and pulmonary toxicity (Figure 6F; Figures S17 and S18) in comparison to the saline group. By contrast, PDS NAs exhibited potent antitumor effectiveness along with favorable therapeutic safety. Additionally, the nano‐cocktail was effective in mitigating hemolytic toxicity associated with SAL (Figure S19). Taking these results together, such a nano‐cocktail, precisely engineered through molecular co‐assembly of a DTX dimer and SAL, demonstrated considerable potential for development into a promising anticancer nanomedicine with high therapeutic efficiency and safety.

Encouraged by the promising synergistic antitumor outcomes of the nano‐cocktail, we further investigated the eradication efficiency of CSCs in vivo. Initially, flow cytometry was performed using two well‐established strategies associated with BCSC phenotypes, including CD133^+^ and CD44^+^CD24^−^. As shown in Figure 6G,H, and Figures S20 and S21, SAL Sol and DS Sol exhibited limited efficacy in inhibiting CSCs owing to their rapid clearance rate and less tumor accumulation in vivo. In contrast, the PDS NAs group demonstrated the most potent anti‐CSCs effect with the lowest percentage of CSCs as a result of the synergistically enhanced anti‐CSCs capacity of SAL. Note that the mice treated with DTX led to a significant increase in CSCs, reflecting that chemotherapy alone not only failed to eliminate highly resistant CSCs, but also enhanced their stemness‐related properties. To validate the excellent performance of nano‐cocktail in eradicating CSCs, we proceeded to perform immunofluorescent staining to assess the expression levels of three core CSC pluripotency factors (Sox2, Oct4, and Nanog) in tumor tissues. Consistently, PDS NAs exhibited a significant downregulation of pluripotency factors associated with CSCs among all groups (Figure S22). The collective data presented above demonstrated that the nano‐cocktail held great potential for eradicating CSCs and effectively counteracting the chemotherapy‐induced tumor stemness upregulation through incorporating SAL.

**FIGURE 7:**
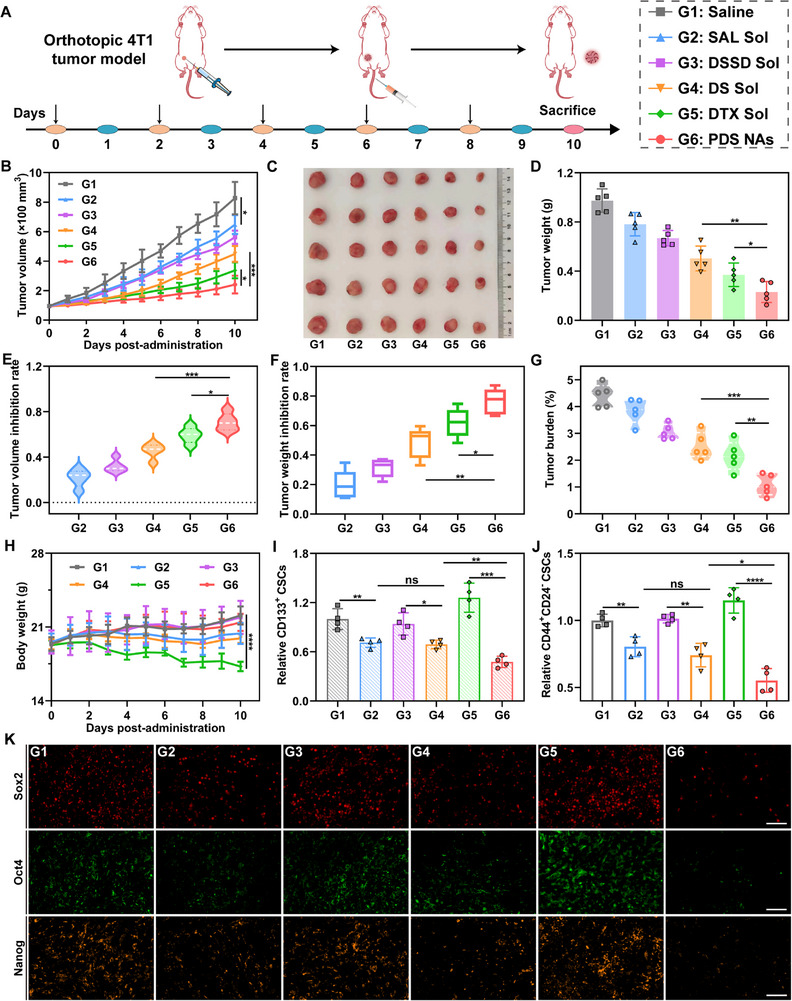
In vivo antitumor efficacy of the nano‐cocktail in mice bearing orthotopic 4T1 tumors. (A) Diagrammatic representation of therapeutic process. (B) Tumor growth curves following different treatments (*n* = 5). (C) Tumor images after the last treatment (*n* = 5). (D) Tumor weight following the completion of treatment (*n* = 5). (E,F) The inhibition rate of tumor volume and weight across different groups (*n* = 5). (G) Tumor burden following the final treatment (*n* = 5). (H) Body weight alterations throughout the treatment period (*n* = 5). (I) Percentage of CD133^+^ CSCs in tumor tissues (*n* = 4). (J) Proportion of CSCs characterized as CD44^+^CD24^−^ in tumor tissues (*n* = 4). (K) Immunofluorescence‐stained images of the tumor sections with three CSC pluripotency factors (Sox2, Oct4, and Nanog). Scale bar represents 50 µm. Data shown as mean ± SD. *p* values: *p < 0.05, **p < 0.01, ***p < 0.001, ****p < 0.0001, ns indicates no significance.

These favorable therapeutic outcomes provided us with a strong motivation to further validate the synergistic antitumor response and CSCs elimination effect of the nano‐cocktail in the orthotopic 4T1 tumor model. For this purpose, we established a mouse model bearing an orthotopic 4T1 tumor, known for its higher malignancy compared to xenograft tumors, and implemented a similar treatment schedule as depicted in Figure 7A. Based on the results from the analysis of tumor volume (Figure 7B; Figure S23), excised tumor image (Figure 7C), and tumor weight (Figure 7D), it could be observed that the nano‐cocktail (PDS NAs) exhibited superior in vivo antitumor efficacy compared to all other experimental groups. This conclusion was further substantiated by the outcomes related to tumor volume and weight inhibition rate as well as tumor burden (Figure 7E–G). Moreover, the remarkable antitumor efficacy of PDS NAs was also confirmed through H&E and TUNEL staining analysis (Figure S24). From the body weight curve, only the mice treated with DTX Sol, SAL Sol, and DS Sol exhibited weight loss, indicating the favorable therapeutic safety of PDS NAs (Figure 5H). Furthermore, serum biochemical analysis (Figure S25) and H&E staining sections of major organs (Figure S26) provide additional evidence supporting the excellent biocompatibility of PDS NAs. We next determined the quantities of CSCs in tumor tissues following treatment with various formulations. Our findings demonstrated that PDS NAs conspicuously decreased the population of CD133^+^ CSCs and CD44^+^CD24^−^ CSCs (Figure 7I,J; Figures S27 and S28), and also downregulated the expression of CSC pluripotency factors within tumors. Together, these data were in accordance with the evaluation outcomes from the 4T1 xenograft tumor model, highlighting the exceptional potency of the nano‐cocktail in eradicating CSCs, thereby amplifying its antitumor effects.

## 3 Discussion

Revealing the underlying mechanisms behind therapy resistance has enabled CSCs to be an important therapeutic target for intervention in cancer treatment [50]. Despite constituting a small subset within tumors, CSCs possess multiple resistance mechanisms against conventional chemotherapeutic agents [13, 17]. Furthermore, chemotherapy acts as a selective stimulus to induce phenotypic changes of non‐stem cancer cells, resulting in the enrichment of drug‐resistant CSCs and consequently mediating tumor regeneration and metastasis [20]. Therefore, it is critical to devise precise strategies for effectively eradicating CSCs in order to advance cancer therapy. Here, we designed a carrier‐free nano‐cocktail based on molecular nanoassembly of a redox‐responsive dimeric prodrug and an anti‐CSCs agent, enabling simultaneous eradication of both cancer cells and CSCs in an efficient and safe manner. We initially synthesized a dimeric prodrug of DTX by conjugating two DTX molecules together through the disulfide bond as a redox‐responsive linkage. Furthermore, SAL was strategically selected as the second component in the nano‐cocktail owing to its widespread recognition as a promising anti‐CSCs agent. Interestingly, the dimeric prodrug was able to easily co‐assemble with SAL to form stable NAs without requiring extra carrier materials. Meanwhile, a minor proportion of DSPE‐PEG_2K_ (20 wt%) was used for surface decoration to enhance the colloidal stability and pharmacokinetic behavior of NAs. It was worth mentioning that the intermolecular forces between DSSD and SAL were responsible for driving the nano‐cocktail formation, where DSPE‐PEG_2K_ only played a crucial role as a PEGylation modifier in the co‐assembly process, rather than serving as a carrier material. Benefiting from the distinct advantages of prodrug strategy and co‐precipitation nanotechnology, PDS NAs demonstrated exceptional performance in terms of co‐loading efficiency and co‐release behavior. In particular, the nano‐cocktail enabled on‐demand prodrug activation and site‐specific drug release under conditions of GSH overexpression in tumor cells and CSCs, effectively circumventing the risk of off‐target superposition toxicity associated with the DTX/SAL combination. More importantly, the integration of SAL within the nanosystem not only significantly reduced the proportion of CSCs, but also synergistically enhanced the selective toxicity of DTX towards both tumor cells and CSCs.

Formulating DSSD and SAL as a “two‐in‐one” nano‐cocktail significantly enhanced the capacity of synchronous co‐delivery, tumor‐specific accumulation, and synergistic tumor eradication with cancer cells/CSCs double‐killing modality. The surface modification of DSSD/SAL NAs with DSPE‐PEG_2K_ resulted in extended systemic circulation time and increased tumor‐site specific accumulation, laying a solid foundation for the implementation of an antitumor strategy with high efficiency and safety. Precise co‐delivery of DSSD and SAL exhibited potential as a double‐killing therapeutic strategy to effectively eliminate cancer cells and CSCs, so as to counter the limitations of insufficient anti‐CSCs monotherapy efficacy and chemotherapy‐induced tumor stemness upregulation. The in vivo antitumor results obtained from both xenograft and orthotopic 4T1 breast cancer mouse models suggested the potency of nano‐cocktail in synergistically suppressing tumor growth and efficiently reducing tumor stemness. Furthermore, the safety assessment revealed that PDS NAs were successful in evading body weight reduction, hepatic function impairment, and pathological tissue changes induced by the DTX/SAL combination throughout the entire therapeutic duration. The favorable treatment safety of PDS NAs could be ascribed to its unique characteristics of on‐demand prodrug activation and site‐specific drug co‐release within the tumor. The nano‐cocktail fabricated by the molecular prodrug/drug nanoassembly strategy with a dual‐killing mechanism capable of simultaneously eradicating a substantial number of tumor cells and drug‐resistant CSCs, has great promise as a synergistic chemotherapy modality towards clinical breast cancer therapy.

## 4 Conclusion

In summary, we successfully developed a one‐stone‐two‐birds nano‐cocktail by exploiting a prodrug strategy and molecular nanoassembly technique to realize synergistic eradication of cancer cells/stem cells. The small nano‐cocktail offered a powerful tool to simultaneously deal with the challenges of chemotherapy‐induced tumor stemness evolution and CSCs‐mediated chemoresistance. Furthermore, it has achieved remarkable efficacy in suppressing tumor stemness and exerting synergistic antitumor effects in both xenograft and orthotopic 4T1 breast cancer mouse models. We demonstrated that the nano‐cocktail not only elicited robust breast cancer cell death through SAL‐synergizing chemotherapy with DSSD, but also dramatically reduced the proportion of BCSCs in tumors. More importantly, the off‐target superposition toxicity of drug combinations can be avoided by on‐demand prodrug activation and site‐specific SAL release from PDS NAs. As expected, the nano‐cocktail strategy achieved robust therapeutic activity against breast tumor‐bearing mice with reduced systemic toxicity. This study presents a simple and feasible paradigm for the rational design of synergistic chemotherapy nanomedicines with high efficacy and safety.

In the future, such a nano‐cocktail is expected to provide a more effective solution for clinical cancer treatment, potentially realizing breast cancer eradication by simultaneously eliminating cancer cells and CSCs. Particularly, the precise depletion of CSCs would make a significant contribution to reducing the recurrence of breast cancer. However, despite the fact that the clinical translation of carrier‐free nanomedicines is not constrained by the difficulties associated with the development of new carrier materials into pharmaceutical excipients, the clinical application of the nano‐cocktail still faces some practical challenges. On the one hand, tumor heterogeneity remains a major challenge for the clinical translation of most nanomedicines, especially those stimuli‐responsive nanosystems. Tumor heterogeneity may not only lead to differences in the activation efficiency of prodrugs but also affect the synergistic efficacy of two drugs. On the other hand, the difference between animal models and human beings represents another major challenge impeding the clinical translation of nanomedicines. Looking ahead, the synergy and safety of the nano‐cocktails need to be further verified in large animals, including primates. Obviously, the clinical translation of such a uniquely engineered nano‐cocktail still has a long way to go. We will persist in our efforts to advance its clinical translation with the hope that it will truly benefit cancer patients in the near future.

## 5 Methods

### 5.1 Materials

SAL was sourced from Aladdin Biochemical Technology Co. Ltd. (Shanghai, China). DTX, DiR, dithiothreitol (DTT), cell culture medium, Hoechst 33342, and 3‐(4,5‐dimethyl‐2‐thiazolyl)‐2,5‐diphenyl‐2H‐tetrazolium bromide (MTT) were procured from Meilun Biotech Co. Ltd. (Dalian, China). The flow cytometry antibodies were provided by BioLegend, Inc. (USA). The Annexin V‐FITC/PI Apoptosis Kit was purchased from Beijing Solarbio Science & Technology Co., Ltd. (Beijing, China). 1,2‐distearoyl‐sn‐glycero‐3‐phosphoethanolamine‐*N*‐[methoxy(polyethylene glycol)‐2000] (DSPE‐PEG_2K_) was obtained from A.V.T. Pharmaceutical Co., Ltd. (Shanghai, China). 4,4'‐Dithiodibutyric acid was purchased from TCI (Shanghai) Development Co. Ltd. (Shanghai, China). 1‐(3‐Dimethylaminopropyl)‐3‐ethylcarbodiimide hydrochloride (EDCI) and 4‐dimethylaminopyridine (DMAP) were supplied by Energy Chemical (Shanghai, China). Ultra‐low attachment 6‐well plates were acquired from Corning (Sigma‐Aldrich, American). All solvents and reagents employed were of analytical or high‐performance liquid chromatography (HPLC) grade.

### 5.2 Cell Culture

4T1 mouse breast cancer cells, MCF‐7 human breast cancer cells, and 3T3 mouse embryonic fibroblast cells were acquired from the Cell Bank of Chinese Academy of Sciences (Shanghai, China), L02 human normal liver cells were purchased from the Cell Bank of Chinese Academy of Sciences (Beijing, China). 4T1 cancer cells and L02 cells were cultured in RPMI‐1640 medium supplemented with 10% fetal bovine serum (FBS) and 1% penicillin‐streptomycin solution. MCF‐7 cancer cells were cultivated in DMEM, whereas 3T3 cells were maintained in DMEM/F12 medium. Both media contained the aforementioned supplements. Meanwhile, all cell lines were maintained at 37°C with 5% CO2.

### 5.3 Animals

All animals were sourced from Changsheng Biotechnology Co., Ltd. (Shenyang, China). Male Sprague–Dawley (SD) rats weighing 180–220 g were used for the pharmacokinetic study, while female BALB/c mice weighing 18–22 g were utilized for the pharmacodynamic study.

### 5.4 Synthesis of a Dimeric Prodrug of DTX

DTX (0.4 mmol) and 4,4'‐dithiodibutyric acid (0.2 mmol) were dissolved in dichloromethane (CH_2_Cl_2_). Subsequently, EDCI (0.8 mmol) and DMAP (0.04 mmol) were sequentially added to the reaction mixture. After stirring for 1 h at 25°C, additional EDCI (0.4 mmol) and DMAP (0.04 mmol) were introduced, and the reaction was permitted to proceed for another 24 h under identical conditions [48]. Purification of the reaction product was achieved via a preparative liquid chromatography system, which yielded DSSD at a rate of 32.6%. To assess the purity of the prodrug, a reverse‐phase HPLC system was employed. The structure of prodrug was verified using MS, ^1^H NMR, and ^13^C NMR.

### 5.5 Investigation of the Optimal Synergistic Dosage Proportion of DSSD and SAL

We initially prepared DSSD/SAL hybrid nanoassemblies (NAs) through the one‐step nanoprecipitation method in order to explore the ideal dosage ratio between DSSD and SAL. Specifically, we separately solubilized DSSD and SAL in a mixture of anhydrous ethanol and tetrahydrofuran, resulting in each solution at a concentration of 5 mg mL^−1^. The DSSD/SAL mixture was subsequently added dropwise to deionized water (2 mL) at molar ratios of 5:1, 4:1, 3:1, 2:1, 1:1, 1:2, 1:3, 1:4, and 1:5, and stirred at 1500 rpm for 2 min to prepare bare DSSD/SAL NAs. Under a vacuum, the organic reagents within the system were eliminated at a temperature of 34°C, after which the preparation volume was readjusted to 2 mL.

Moreover, the synergistic effects of DSSD and SAL on tumor cells were evaluated through the MTT assay. Briefly, 4T1 cancer cells (2 × 10^3^ cells/well) were seeded into 96‐well plates and cultured until adherent. The initial medium was replaced with fresh medium supplemented with varying concentrations of DSSD and SAL at molar ratios that ranged from 5:1 to 1:5, respectively. After 48 h of incubation, each well received an addition of MTT solution (20 µL), followed by an additional 4 h incubation. Afterwards, the liquid in the wells was carefully removed and replaced with dimethyl sulfoxide (DMSO, 200 µL) for the dissolution of formed formazan crystals. Finally, cell viability was determined at 490 nm utilizing a microplate reader (Thermo Scientific, USA). The cooperativity index (CI) was computed to evaluate the synergistic effect between DSSD and SAL. It was calculated as follows:

$$\begin{array}{cc} \mathrm{CI} & =A\times IC_{50}\left(\mathrm{DSSD}/\mathrm{SAL}\right)/IC_{50}\left(\mathrm{DSSD}\right)\\ & \;+B\times IC_{50}\left(\mathrm{DSSD}/\mathrm{SAL}\right)/IC_{50}\left(\mathrm{SAL}\right) \end{array}$$

where “*A*” and “*B*” represented the dosage proportion of DSSD and SAL when DSSD and SAL were treated collectively; IC_50_ (DSSD) or IC_50_ (SAL) indicated the calculated IC_50_ upon treatment of cells with DSSD or SAL, respectively; IC_50_ (DSSD/SAL) referred to the calculated IC_50_ when DSSD was combined with SAL at different molar ratios. The results could be categorized as antagonistic (CI > 1), additive (CI = 1), and synergistic (CI < 1) effects.

### 5.6 Preparation and Characterization of DSSD/SAL NAs

We fabricated the optimal DSSD/SAL NAs (DS NAs) utilizing the one‐step nanoprecipitation technique at a 1:1 molar ratio. Furthermore, the PEGylated NAs were prepared with the addition of different concentrations of DSPE‐PEG_2K_ (10 wt%, 20 wt%, and 30 wt%). The formulation containing 20 wt% DSPE‐PEG_2K_ was used for the final preparation of PEGylated DSSD/SAL NAs (PDS NAs) via a similar preparation process. Particle diameter, zeta potential, and PDI of both DS NAs and PDS NAs were measured using a Zetasizer (Nano ZS, Malvern Co., UK). Additionally, the morphologies of NAs were visualized through TEM (Hitachi, HT7700, Japan) after staining with 2% phosphotungstic acid. The drug loading (DL) rates of DS NAs and PDS NAs were determined as follows (1)–(4):

(1) $$\begin{array}{cc} DL_{\mathrm{DTX}}\;\text{of DS NAs}\; & =m_{\mathrm{DSSD}}\times M_{\mathrm{DTX}}\times 2/M_{\mathrm{DSSD}}/\left(m_{\mathrm{DSSD}}+m_{\mathrm{SAL}}\right)\\ & \;\times 100\% \end{array}$$

(2) $$DL_{\mathrm{SAL}}\;\text{of DS NAs}=m_{\mathrm{SAL}}/\left(m_{\mathrm{DSSD}}+m_{\mathrm{SAL}}\right)\times 100\%$$

(3) $$\begin{array}{cc} DL_{\mathrm{DTX}}\;\mathrm{of}\;\mathrm{PDS}\;\mathrm{NAs} & =0.8\times m_{\mathrm{DSSD}}\times M_{\mathrm{DTX}}\\ & \;\times 2/M_{\mathrm{DSSD}}/\left(m_{\mathrm{DSSD}}+m_{\mathrm{SAL}}\right)\times 100\% \end{array}$$

(4) $$DL_{\mathrm{SAL}}\;\mathrm{of}\;\mathrm{PDS}\;\mathrm{NAs}=0.8\times m_{\mathrm{SAL}}/\left(m_{\mathrm{DSSD}}+m_{\mathrm{SAL}}\right)\times 100\%$$

where *m*_DSSD_ is the total mass of DSSD in the formulation, *m*_SAL_ is the total mass of SAL in the formulation, *M*_DTX_ is the molecular weight of DTX, *M*_DSSD_ is the molecular weight of DSSD, and *M*_SAL_ is the molecular weight of SAL.

### 5.7 Colloidal Stability

The stability of the NAs was evaluated by measuring particle size variations. Specifically, both DS NAs and PDS NAs were incubated at a concentration of 0.5 mg mL^−1^ in PBS (pH 7.4) for 12 h at 37°C. Particle diameters were measured at 0, 0.5, 1, 2, 4, 6, 8, 10, and 12 h using a Zetasizer. Stability of PDS NAs in PBS (pH 7.4) containing 10% FBS was further investigated at various time intervals. Furthermore, we evaluated the long‐term storage stability of PDS NAs by storing them for 30 days at 4°C. To examine the lyophilization stability of PDS NAs, 10% sucrose was used as a protective agent. The stability of lyophilized samples was characterized by recording the variations in particle diameter before and after lyophilization. Additionally, the morphological characteristics of the freeze‐dried PDS NAs were evaluated through TEM.

### 5.8 Exploration of Co‐Assembly Mechanism of DSSD and SAL

The co‐assembly mechanism between DSSD and SAL was investigated via molecular docking simulations conducted on the Yinfo Cloud Platform. Using the compound structure preparation utility, the 3D models of DSSD and SAL were initially generated and then optimized through energy minimization in the MMFF94 force field. Afterward, small‐molecule‐small‐molecule docking was carried out with the AutoDock Vina program. A semi‐flexible docking approach was employed to obtain as many as nine output conformations after an internal clustering process, allowing for a detailed analysis of the interactions [29]. Furthermore, different intermolecular interaction disruptors were used to co‐incubate with NAs in order to further explore the co‐assembly driving forces. Briefly, DS NAs were suspended in a solution containing NaCl, SDS, and urea, with a concentration of 10 mM, then incubated at 37°C for 12 h. The Zetasizer was used to determine the particle diameter variations of the DS NAs.

### 5.9 Reduction‐Responsive Drug Release and Nanoassembly Collapse

The DTX release profiles from PDS NAs were evaluated using a medium composed of PBS (pH 7.4) with 30% anhydrous ethanol, with DTT as the reduction‐stimulus agent. Specifically, 1 mL of PDS NAs was added to the release medium (30 mL) containing varying concentrations of DTT (0, 1, 2, and 5 mM), and the mixture was incubated at 37°C. At 1, 2, 4, 6, 8, and 12 h, 200 µL samples were taken out and immediately replaced with fresh release medium. Quantification of the cumulative DTX release from PDS NAs was performed using HPLC. Furthermore, PDS NAs were incubated in a release medium containing 5 mM DTT. Alterations in their morphology and particle size were monitored utilizing TEM and Zetasizer.

### 5.10 Cellular Uptake

4T1 cancer cells (5 × 10^4^ cells/mL) were seeded onto glass coverslips in 12‐well plates. After a 24‐h cultivation period, free coumarin‐6 solution (C‐6 Sol) or C‐6‐labeled PDS NAs (C‐6/PDS NAs), both at 200 ng mL^−1^ C‐6 concentration, were added for 0.5 or 2 h. After washing the cells three times with cold PBS, they were fixed using 4% paraformaldehyde, and the nuclei were labeled with Hoechst 33342 for 10 min. The intracellular fluorescence was observed by means of CLSM (C2, Nikon, Japan). For evaluation by flow cytometry, 4T1 cancer cells (1 × 10^5^ cells/well) were plated into 12‐well plates for 24 h. After being treated with C‐6 Sol or C‐6/PDS NAs, the cells underwent washing, harvesting, and resuspension in cold PBS for subsequent quantitative evaluation. Untreated cells served as the control.

### 5.11 Cytotoxicity Assay

To assess the in vitro antiproliferative efficacy of PDS NAs, the MTT assay was conducted on two types of cancer cell lines (4T1 and MCF‐7 cells) and two normal cell lines (L02 and 3T3 cells). Briefly, 4T1 and MCF‐7 cancer cells (2 × 10^3^ cells per well) were seeded into 96‐well plates and allowed to adhere. Fresh media consisting of different concentrations of SAL solution (SAL Sol), DSSD solution (DSSD Sol), DSSD/SAL solution (DS Sol), DTX solution (DTX Sol), and PDS NAs were subsequently added to the cells. A negative control was established by culturing cells in fresh blank medium. Cell viability was evaluated by MTT assay following a 48‐h culture period, as outlined in prior studies. In addition, the cytotoxic effects of SAL Sol, DSSD Sol, DS Sol, DTX Sol, and PDS NAs towards normal cells were also assessed using L02 and 3T3 cells through the same experimental procedure.

### 5.12 Apoptosis Assay

4T1 cancer cells (1 × 10^5^ cells per well) were plated in 12‐well plates and cultured overnight. Subsequently, the existing medium was exchanged for a fresh medium that contained SAL Sol, DSSD Sol, DS Sol, DTX Sol, and PDS NAs. After 24 h of incubation, cell samples were collected by EDTA‐free trypsin digestion and labeled with Annexin V‐FITC and PI following the manufacturer's protocol. Finally, flow cytometry was applied to assess the apoptosis rate, followed by data analysis utilizing FlowJo software.

### 5.13 Formation and Identification of CSCs

The serum‐free suspension culture method was employed to enrich CSCs. In brief, 4T1 cancer cells (1 × 10^4^ cells/mL) were introduced into 6‐well ultralow attachment plates (Corning) with DMEM/F12 culture medium, supplemented with 1 × B27 supplement (Gibco), basic fibroblast growth factor (bFGF, 20 ng mL^−1^), epidermal growth factor (EGF, 20 ng mL^−1^), 0.4% bovine serum albumin, insulin (5 µg mL^−1^), and 1% penicillin‐streptomycin solution. In the absence of serum, the cells formed 3D mammospheres with a spherical structure. Half of the medium in each well was replaced every 3–4 days with fresh medium to partially refresh the environment. After 7–10 days of culture, the CSCs‐enriched 3D mammosphere model was successfully established and subsequently utilized for identification and further experimental investigations.

In order to identify CSCs, the 3D mammospheres were harvested through gentle centrifugation, followed by digestion into single cells using StemPro Accutase (Invitrogen). Afterwards, CSCs were screened with the APC‐labeled anti‐mouse CD133 antibody, or FITC‐conjugated anti‐mouse/human CD44 and PE‐conjugated anti‐mouse CD24 antibodies under dark conditions at 4°C. After being washed and resuspended in cold PBS, flow cytometry was employed to evaluate the expression of CD133, as well as CD44 and CD24, in 3D mammospheres.

### 5.14 GSH Level Detection

To detect the levels of GSH, 4T1 cancer cells or CSCs were rinsed with cold PBS, harvested by centrifugation, and then intracellular GSH levels were assessed according to the protocol provided in the GSH assay kit.

### 5.15 In Vitro Anti‐CSCs Efficacy

4T1 cancer cells (2 × 10^5^ cells per well) were plated in 6‐well plates and allowed to adhere. Subsequently, we assessed the impact of SAL on CSCs by incubating the cells with SAL and conducting cell staining using CD133^+^ and CD44^+^CD24^−^ surface markers. The staining procedure was carried out following the aforementioned protocol. In addition, we investigated the CD133^+^ and CD44^+^CD24^−^ populations on 3D mammospheres after treatment with different formulations. In brief, the dissociated mammospheres (2 × 10^5^ cells/well) were plated into 6‐well ultralow attachment plates and incubated overnight. Afterward, the cells were treated with different formulations and cultured for 48 h. Relevant to the established procedure, the cells were stained and evaluated using flow cytometry to determine the percentage of CD133^+^ CSCs and CD44^+^CD24^−^ CSCs.

### 5.16 Pharmacokinetics

DiR was selected as a fluorescent probe for labeling PDS NAs, referred to as DiR/PDS NAs. Male SD rats were given DiR Sol or DiR/PDS NAs via intravenous injection at a dose of 1 mg kg^−1^ DiR (*n* = 6). At predesigned time points (0.033, 0.083, 0.25, 0.5, 1, 2, 4, 8, 12, and 24 h), blood samples were collected and plasma was obtained by centrifuging at 10000 rpm for 3 min. The plasma samples were processed using the protein precipitation method for the extraction of DiR. Finally, DiR concentrations in plasma were quantified by microplate reader (Ex/Em = 748/780 nm).

### 5.17 Biodistribution

For the establishment of a 4T1 mammary tumor model, female BALB/c mice received subcutaneous injections of 4T1 cancer cells in the right flank. When the tumor size reached approximately 400 mm^3^, a random assignment was performed to divide the mice into two groups (*n* = 3). DiR Sol or DiR/PDS NAs were intravenously injected into the mice at a dose of 0.8 mg kg^−1^ of DiR. At 1, 2, 4, 6, 8, 12, and 24 h, the mice were anesthetized and subjected to imaging with the IVIS. 24 h after injection, mice were euthanized, and fluorescence imaging was conducted on collected samples of major organs and tumors. Fluorescence signals were quantified by the ROI tool (*n* = 3).

### 5.18 In Vivo Antitumor and Anti‐CSCs Efficacy

To evaluate the antitumor potency of PDS NAs, we established both xenograft and orthotopic 4T1 tumor models using BALB/c mice. Once the volume of the tumor was approximately 100 mm^3^, we randomized the mice into six groups (*n* = 5). Then the mice received intravenous injections of saline, SAL Sol, DSSD Sol, DS Sol, DTX Sol, and PDS NAs with each dose equivalent to 5 mg kg^−1^ of DTX, on alternate days. Daily measurements were taken to record changes in tumor volume and body weight. Upon completion of the observation period, blood samples from mice in each group were obtained and then centrifuged to obtain serum samples for evaluating hepatic and renal function. Mice were euthanized, after which their major organs and tumors were isolated and collected. Subsequently, the tumor tissues were photographed and weighed. Main organs and tumors were preserved in a 4% paraformaldehyde solution prior to undergoing H&E staining for the assessment of pathological alterations. Ki67 immunohistochemistry and TUNEL immunofluorescence staining were applied to evaluate the proliferation rates and apoptosis levels of tumor cells. Furthermore, immunofluorescence staining was also used to assess the expression of CSC pluripotency factors (Sox2, Oct4, and Nanog) in tumor tissues. The tumor burden and tumor inhibitory rate were calculated as follows (5)–(7):

(5) $$\mathrm{Tumor}\;\mathrm{burden}\left(\%\right)=\left(W_{\mathrm{tumor}}/W_{\mathrm{mice}}\right)\times 100\%$$

where *W*_tumor_ represents the tumor mass and *W*_mice_ refers to the body weight of the mice.

(6) $$\mathrm{Tumor}\;\mathrm{volume}\;\mathrm{inhibition}\;\mathrm{rate}=\left(V_{c}-V_{e}\right)/V_{c}$$

where *V*_c_ denotes the tumor volume in the control (saline) group and *V*_e_ indicates the tumor volume in each respective experimental group.

(7) $$\mathrm{Tumor}\;\mathrm{weight}\;\mathrm{inhibition}\;\mathrm{rate}=\left(W_{c}-W_{e}\right)/W_{c}$$

where *W*_c_ denotes the tumor weight in the control (saline) group and *W*_e_ indicates the tumor weight in each respective experimental group.

### 5.19 Flow Cytometry Analysis

The tumor samples were obtained from mice, and CSCs within the tumor tissues were identified by CD133^+^ and CD44^+^CD24^−^ cell surface marker staining. Briefly, tumors were dissected into small pieces, subjected to collagenase digestion, followed by filtration through a membrane filter (pore size, 70 µm) to produce suspensions of single cells. Afterward, the cells were incubated with the corresponding antibodies as per the previously mentioned protocol and examined by flow cytometry to quantify the proportion of CSCs in tumor tissues.

### 5.20 Hemolysis Assay

Next, we evaluated the compatibility of PDS NAs by means of hemolysis tests. Fresh blood was obtained from healthy rats and placed in heparin‐coated anticoagulant tubes. To extract erythrocytes, the supernatant was isolated from the blood sample through centrifugation at 8000 rpm for 5 min, followed by three rinses with saline solution. A 2% suspension of erythrocytes was prepared and co‐incubated with SAL Sol and PDS NAs for 1 and 3 h, respectively. Afterwards, the samples underwent centrifugation (5000 rpm, 5 min), after which 200 µL of the supernatant was carefully transferred to a 96‐well microplate. The absorbance measurements were conducted at 545 nm using a microplate reader. Positive and negative controls consisted of water and saline, respectively.

### 5.21 Statistical Analysis

All data were presented as the mean ± SD. Statistical differences were analyzed using two‐tailed Student's *t*‐test. Statistical significance was set at *p* < 0.05, where *p < 0.05, **p < 0.01, ***p < 0.001, and ****p < 0.0001.

## Author Contributions

C.L., Y.C., and T.Z. conceived this work. T.Z. and Y.C. prepared and characterized the materials. T.Z., Y.C., H.Y., S.Y., and H.Z. carried out the in vitro, ex vivo and in vivo experiments. Y.W., S.Z., and Q.C. contributed technical support. T.Z., C.L., Y.C., H.Y., J.S., and Z.H. analyzed the data and discussed the results. T.Z., C.L., and Y.C. wrote and edited the manuscript. C.L. supervised the project.

## Conflicts of Interest

The authors declare no conflicts of interest.

## Ethics Statement

All experiments involving animals complied with the Guidelines for Care and Use of Laboratory Animals of Shenyang Pharmaceutical University and received approval from the university's Animal Ethics Committee. The approved protocol number for the laboratory is SYPU‐IACUC‐2022‐0302‐045.

## Supporting information




**Supporting file 1**: exp270106‐sup‐0001‐SuppMat.docx.

## Data Availability

All necessary data for the evaluation of the conclusions drawn in this paper are available in the paper and/or the Supporting Information.

## References

[1] R. L. Siegel , K. D. Miller , N. S. Wagle , and A. Jemal , “Cancer Statistics, 2023,” CA: A Cancer Journal for Clinicians 73 (2023): 17. 36633525 10.3322/caac.21763

[2] Y. Song , Y. Du , C. Hu , et al., “Metformin‐Mediated Immunosuppressive Microenvironment Remodeling in Combination with Chemotherapy via a Spatial‐Specific Multi‐Responsive Carrier‐Free Self‐Assembled Nanoparticle,” Advanced Functional Materials 34 (2024): 202316145.

[3] S. Theivendran , S. Lazarev , and C. Yu , “Mesoporous Silica/Organosilica Nanoparticles for Cancer Immunotherapy,” Exploration (Beijing, China) 3 (2023): 20220086. 37933387 10.1002/EXP.20220086 PMC10624378

[4] Z. Fu , S. Li , S. Han , C. Shi , and Y. Zhang , “Antibody Drug Conjugate: The “Biological Missile” for Targeted Cancer Therapy,” Signal Transduction and Targeted Therapy 7 (2022): 93, 10.1038/s41392-022-00947-7. 35318309 PMC8941077

[5] U. Anand , A. Dey , A. K. S. Chandel , et al., “Cancer Chemotherapy and Beyond: Current Status, Drug Candidates, Associated Risks and Progress in Targeted Therapeutics,” Genes & Diseases 10 (2023): 1367–1401, 10.1016/j.gendis.2022.02.007. 37397557 PMC10310991

[6] R. Lin , J. Yan , B. Gong , et al., “Self‐Delivered Transformable Nanosystem Capable of Enhancing Photodynamic Effectiveness and Multi‐Target Ameliorating Immunosuppression for Treatment of Breast Cancer and Lung Metastasis,” Advanced Functional Materials 34, no. 42 (2024): 2405051.

[7] F. Zhou , L. Huang , S. Li , et al., “From Structural Design to Delivery: mRNA Therapeutics for Cancer Immunotherapy,” Exploration (Beijing, China) 4 (2024): 20210146. 38855617 10.1002/EXP.20210146 PMC11022630

[8] F. Yang , S. Li , Q. Ji , et al., “Highly Efficient Printed Quantum Dot Light‐Emitting Diodes Through Ultrahigh‐Definition Double‐Layer Transfer Printing,” Advanced Science 11 (2024): 2309786.38760898

[9] T. Reya , S. J. Morrison , M. F. Clarke , and I. L. Weissman , “Stem Cells, Cancer, and Cancer Stem Cells,” Nature 414 (2001): 105–111, 10.1038/35102167. 11689955

[10] C. T. Jordan , M. L. Guzman , and M. Noble , “Cancer Stem Cells,” New England Journal of Medicine 355 (2006): 1253–1261, 10.1056/NEJMra061808. 16990388

[11] T. Lapidot , C. Sirard , J. Vormoor , et al., “A Cell Initiating Human Acute Myeloid Leukaemia After Transplantation Into SCID Mice,” Nature 367 (1994): 645–648, 10.1038/367645a0. 7509044

[12] J. P. Medema , “Cancer Stem Cells: The Challenges Ahead,” Nature Cell Biology 15 (2013): 338–344, 10.1038/ncb2717. 23548926

[13] T. Chiba , A. Kamiya , O. Yokosuka , and A. Iwama , “Cancer Stem Cells in Hepatocellular Carcinoma: Recent Progress and Perspective,” Cancer Letters 286 (2009): 145–153, 10.1016/j.canlet.2009.04.027. 19464789

[14] J. D. Lathia , S. C. Mack , E. E. Mulkearns‐Hubert , C. L. Valentim , and J. N. Rich , “Cancer Stem Cells in Glioblastoma,” Genes & Development 29 (2015): 1203–1217, 10.1101/gad.261982.115. 26109046 PMC4495393

[15] Z. Mirza and S. Karim , “Nanoparticles‐Based Drug Delivery and Gene Therapy for Breast Cancer: Recent Advancements and Future Challenges,” Seminars in Cancer Biology 69 (2021): 226–237. 31704145 10.1016/j.semcancer.2019.10.020

[16] G. Bianchini , J. M. Balko , I. A. Mayer , M. E. Sanders , and L. Gianni , “Triple‐Negative Breast Cancer: Challenges and Opportunities of a Heterogeneous Disease,” Nature Reviews Clinical Oncology 13 (2016): 674–690, 10.1038/nrclinonc.2016.66. PMC5461122 27184417

[17] X. Bai , J. Ni , J. Beretov , P. Graham , and Y. Li , “Cancer Stem Cell in Breast Cancer Therapeutic Resistance,” Cancer Treatment Reviews 69 (2018): 152–163, 10.1016/j.ctrv.2018.07.004. 30029203

[18] J. Guan , Y. Wu , X. Liu , et al., “A Novel Prodrug and Its Nanoformulation Suppress Cancer Stem Cells by Inducing Immunogenic Cell Death and Inhibiting Indoleamine 2, 3‐dioxygenase,” Biomaterials 279 (2021): 121180, 10.1016/j.biomaterials.2021.121180. 34768152

[19] T. Shibue and R. A. Weinberg , “EMT, CSCs, and Drug Resistance: The Mechanistic Link and Clinical Implications,” Nature Reviews Clinical Oncology 14 (2017): 611–629, 10.1038/nrclinonc.2017.44. PMC5720366 28397828

[20] X. Jiang , J. Liu , J. Mao , et al., “Pharmacological Ascorbate Potentiates Combination Nanomedicines and Reduces Cancer Cell Stemness to Prevent Post‐Surgery Recurrence and Systemic Metastasis,” Biomaterials 295 (2023): 122037, 10.1016/j.biomaterials.2023.122037. 36773429 PMC9998353

[21] L. Yang , P. Shi , G. Zhao , et al., “Targeting Cancer Stem Cell Pathways for Cancer Therapy,” Signal Transduction and Targeted Therapy 5 (2020): 8, 10.1038/s41392-020-0110-5. 32296030 PMC7005297

[22] W.‐D. Wang , Y.‐Y. Guo , Z.‐L. Yang , G.‐L. Su , and Z.‐J. Sun , “Sniping Cancer Stem Cells with Nanomaterials,” ACS Nano 17 (2023): 23262–23298. 38010076 10.1021/acsnano.3c07828

[23] S. Shen , J.‐X. Xia , and J. Wang , “Nanomedicine‐Mediated Cancer Stem Cell Therapy,” Biomaterials 74 (2016): 1–18, 10.1016/j.biomaterials.2015.09.037. 26433488

[24] S. Shen , X. Xu , S. Lin , et al., “A Nanotherapeutic Strategy to Overcome Chemotherapeutic Resistance of Cancer Stem‐Like Cells,” Nature Nanotechnology 16 (2021): 104–113, 10.1038/s41565-020-00793-0. 33437035

[25] P. B. Gupta , T. T. Onder , G. Jiang , et al., “Identification of Selective Inhibitors of Cancer Stem Cells by High‐Throughput Screening,” Cell 138 (2009): 645–659, 10.1016/j.cell.2009.06.034. 19682730 PMC4892125

[26] D. Qi , Y. Liu , J. Li , J. H. Huang , X. Hu , and E. Wu , “Salinomycin as a Potent Anticancer Stem Cell Agent: State of the Art and Future Directions,” Medicinal Research Reviews 42 (2022): 1037–1063, 10.1002/med.21870. 34786735 PMC9298915

[27] X. Huang , B. Borgström , J. Stegmayr , et al., “The Molecular Basis for Inhibition of Stemlike Cancer Cells by Salinomycin,” ACS Central Science 4 (2018): 760–767, 10.1021/acscentsci.8b00257. 29974072 PMC6026786

[28] Y. Chen , T. Zhao , M. Bai , et al., “Emerging Small Molecule‐Engineered Hybrid Nanomedicines for Cancer Therapy,” Chemical Engineering Journal 435 (2022): 135160, 10.1016/j.cej.2022.135160.

[29] X. Sun , S. Zhang , P. Wang , et al., “A Molecularly Tailored Closed‐Loop Tumor Cell Energy Nanodepleter for Cancer Starvation Therapy,” Nano Today 57 (2024): 102374, 10.1016/j.nantod.2024.102374.

[30] Y. Cui , M. Zhao , Y. Yang , et al., “Reversal of Epithelial‐Mesenchymal Transition and Inhibition of Tumor Stemness of Breast Cancer Cells Through Advanced Combined Chemotherapy,” Acta Biomaterialia 152 (2022): 380–392, 10.1016/j.actbio.2022.08.024. 36028199

[31] Z. Zhang , Q. Deng , C. Xiao , Z. Li , and X. Yang , “Rational Design of Nanotherapeutics Based on the Five Features Principle for Potent Elimination of Cancer Stem Cells,” Accounts of Chemical Research 55 (2022): 526–536, 10.1021/acs.accounts.1c00635. 35077133

[32] Z. Wang , S. Zhang , Z. Kong , et al., “Self‐Adaptive Nanoassembly Enabling Turn‐on Hypoxia Illumination and Periphery/Center Closed‐Loop Tumor Eradication,” Cell Reports Medicine 4 (2023): 101014, 10.1016/j.xcrm.2023.101014. 37075700 PMC10140616

[33] H. Zhang , J. Wang , H. Wu , et al., “On‐Site Self‐Penetrating Nanomedicine Enabling Dual‐Priming Drug Activation and Inside‐Out Thrombus Ablation,” ACS Nano 18 (2024): 34683–34697, 10.1021/acsnano.4c09986. 39665339

[34] H. Zhang , S. Zhang , S. Liu , et al., “Self‐Actuated Clot‐Piercing Nanoassembly Enabling Adaptable Drug Activation and Synergistic Thrombus Ablation,” Advanced Functional Materials 2025, no. 35 (2025): 2416968, 10.1002/adfm.202416968.

[35] Y. Wang , P. Wang , W. Li , et al., “Cascaded Multiresponsive Supramolecular Dimer‐Engineered Nano‐Modulator Enabling Spatiotemporally Adaptable Tumor Immune Microenvironment Remodeling in Photodynamic Immunotherapy,” Nano Today 56 (2024): 102305, 10.1016/j.nantod.2024.102305.

[36] H. Zhang , J. Wang , R. Han , B. Sun , and C. Luo , “Bioorthogonal Chemistry‐Driven Anticancer Nanotherapeutics,” Trends in Chemistry 5 (2023): 697–710, 10.1016/j.trechm.2023.05.006.

[37] Z. Lin , Y. Wang , W. Li , et al., “A Natural Compound‐Empowered Podophyllotoxin Prodrug Nanoassembly Magnifies Efficacy‐Toxicity Benefits in Cancer Chemotherapy,” Asian Journal of Pharmaceutical Sciences 19 (2024): 100892, 10.1016/j.ajps.2024.100892. 39246509 PMC11374962

[38] S. Zhang , J. Guan , M. Sun , et al., “Self‐Delivering Prodrug‐Nanoassemblies Fabricated by Disulfide Bond Bridged Oleate Prodrug of Docetaxel for Breast Cancer Therapy,” Drug Delivery 24 (2017): 1460–1469, 10.1080/10717544.2017.1381201. 28950729 PMC8241025

[39] X. Zhang , N. Li , S. Zhang , et al., “Emerging Carrier‐Free Nanosystems Based on Molecular Self‐Assembly of Pure Drugs for Cancer Therapy,” Medicinal Research Reviews 40 (2020): 1754–1775, 10.1002/med.21669. 32266734

[40] S. Li , X. Shan , Y. Wang , et al., “Dimeric Prodrug‐Based Nanomedicines for Cancer Therapy,” Journal of Controlled Release 326 (2020): 510–522, 10.1016/j.jconrel.2020.07.036. 32721523

[41] C. Wang , S. Li , B. Qian , et al., “AIEgen‐Functionalized Nanoprobes and Nanomedicines for Cancer Diagnosis and Therapy,” Coordination Chemistry Reviews 520 (2024): 216148, 10.1016/j.ccr.2024.216148.

[42] F. Yang , Q. Ji , R. Liao , et al., “Precisely Engineering a Dual‐Drug Cooperative Nanoassembly for Proteasome Inhibition‐Potentiated Photodynamic Therapy,” Chinese Chemical Letters 33 (2022): 1927–1932, 10.1016/j.cclet.2021.11.056.

[43] R. Liu , C. Luo , Z. Pang , et al., “Advances of Nanoparticles as Drug Delivery Systems for Disease Diagnosis and Treatment,” Chinese Chemical Letters 34 (2023): 107518, 10.1016/j.cclet.2022.05.032.

[44] H. Zhang , Z. Zhao , S. Sun , et al., “Molecularly Self‐Fueled Nano‐Penetrator for Nonpharmaceutical Treatment of Thrombosis and Ischemic Stroke,” Nature Communications 14 (2023): 255, 10.1038/s41467-023-35895-5. PMC9845202 36650139

[45] B. Sun , C. Luo , H. Yu , et al., “Disulfide Bond‐Driven Oxidation‐ and Reduction‐Responsive Prodrug Nanoassemblies for Cancer Therapy,” Nano Letters 18 (2018): 3643–3650, 10.1021/acs.nanolett.8b00737. 29726685

[46] C. Luo , J. Sun , D. Liu , et al., “Self‐Assembled Redox Dual‐Responsive Prodrug‐Nanosystem Formed by Single Thioether‐Bridged Paclitaxel‐Fatty Acid Conjugate for Cancer Chemotherapy,” Nano Letters 16 (2016): 5401–5408, 10.1021/acs.nanolett.6b01632. 27490088 PMC5541379

[47] Y. Wang , C. Luo , S. Zhou , et al., “Investigating the Crucial Roles of Aliphatic Tails in Disulfide Bond‐Linked Docetaxel Prodrug Nanoassemblies,” Asian Journal of Pharmaceutical Sciences 16 (2021): 643–652, 10.1016/j.ajps.2021.02.001. 34849169 PMC8609389

[48] S. Zuo , B. Sun , Y. Yang , et al., “Probing the Superiority of Diselenium Bond on Docetaxel Dimeric Prodrug Nanoassemblies: Small Roles Taking Big Responsibilities,” Small 16 (2020): 2005039, 10.1002/smll.202005039. 33078579

[49] J. J. Rennick , A. P. Johnston , and R. G. Parton , “Key Principles and Methods for Studying the Endocytosis of Biological and Nanoparticle Therapeutics,” Nature Nanotechnology 16 (2021): 266–276, 10.1038/s41565-021-00858-8. 33712737

[50] H.‐M. Zhou , J.‐G. Zhang , X. Zhang , and Q. Li , “Targeting Cancer Stem Cells for Reversing Therapy Resistance: Mechanism, Signaling, and Prospective Agents,” Signal Transduction and Targeted Therapy 6 (2021): 62, 10.1038/s41392-020-00430-1. 33589595 PMC7884707

