# Supplementary material for: One‐Stone‐Two‐Birds Carrier‐Free Nano‐Cocktail Enables Synergistic Eradication of Cancer Cells/Stem Cells in Breast Cancer Treatment
Source: Exploration (Beijing). 2025 Dec 22;6(1):20240259. doi: 10.1002/EXP.20240259 (PMC12970156; doi:10.1002/EXP.20240259)
Supplement: Supplementary file 1 — Supporting file 1: exp270106‐sup‐0001‐SuppMat.docx. [file EXP2-6-20240259-s001.docx]

**Supporting information**

**One-stone-two-birds Carrier-free Nano-cocktail Enables Synergistic Eradication of Cancer Cells/Stem Cells in Breast Cancer Treatment**

*Tongyao Zhao^1#^, Yao Chen^1#^, Haimeng Yuan^1^, Shuqian Yang^1^, Hongyuan Zhang^1^, Yuequan Wang^1^, Shenwu Zhang^1^, Qin Chen^3^, Jin Sun^1,2^, Zhonggui He^1,2^, Cong Luo^1,2*^*

^1^Department of Pharmaceutics, Wuya College of Innovation, Shenyang Pharmaceutical University, Shenyang 110016, P.R. China

^2^Joint International Research Laboratory of Intelligent Drug Delivery Systems, Ministry of Education, Shenyang Pharmaceutical University, Shenyang 110016, P.R. China

^3^Department of Pharmacy, Cancer Hospital of China Medical University, Liaoning Cancer Hospital & Institute, Shenyang 110042, P.R. China

**^#^** These authors contributed equally to this work.

^*^ Corresponding author: Prof. Cong Luo PhD. E-mail: luocong@syphu.edu.cn


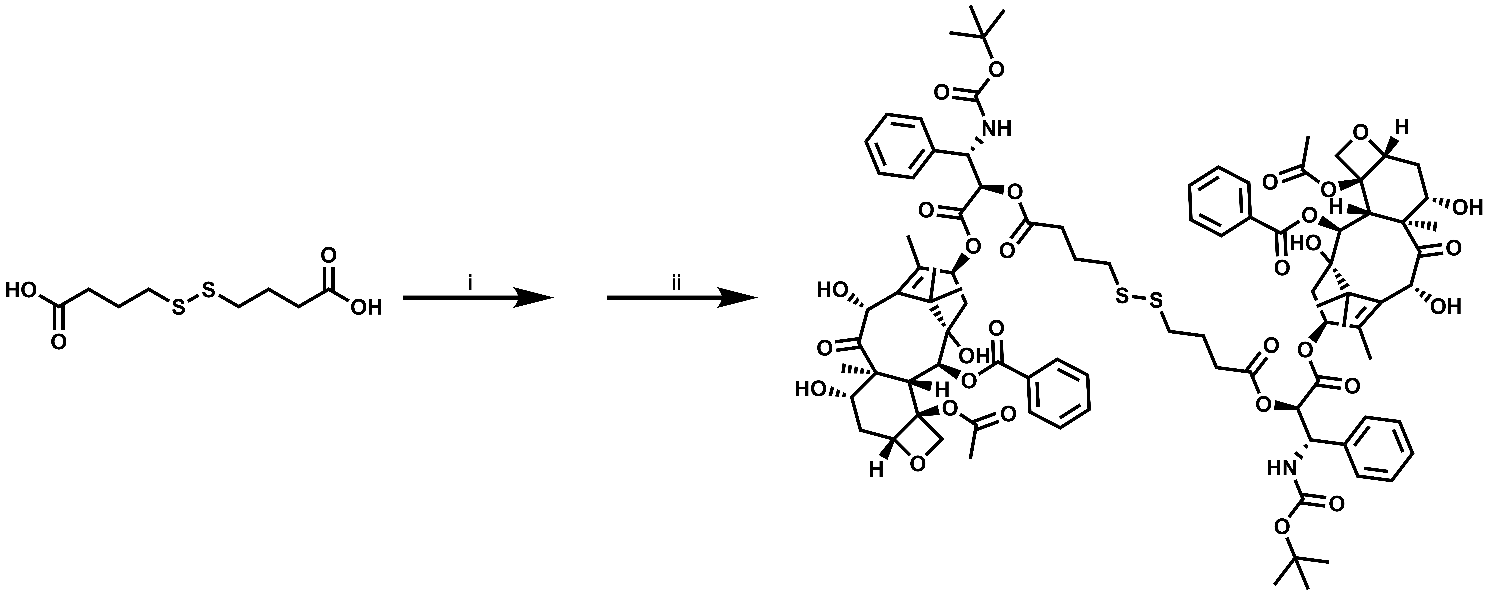


Figure S1. The synthetic route of the dimeric prodrug of docetaxel (DTX-SS-DTX). Reagents and conditions: (i) EDCI, DMAP, DTX, CH_2_Cl_2_, 25 °C, 1 h; (ii) EDCI, DMAP, 25 °C, 24 h.


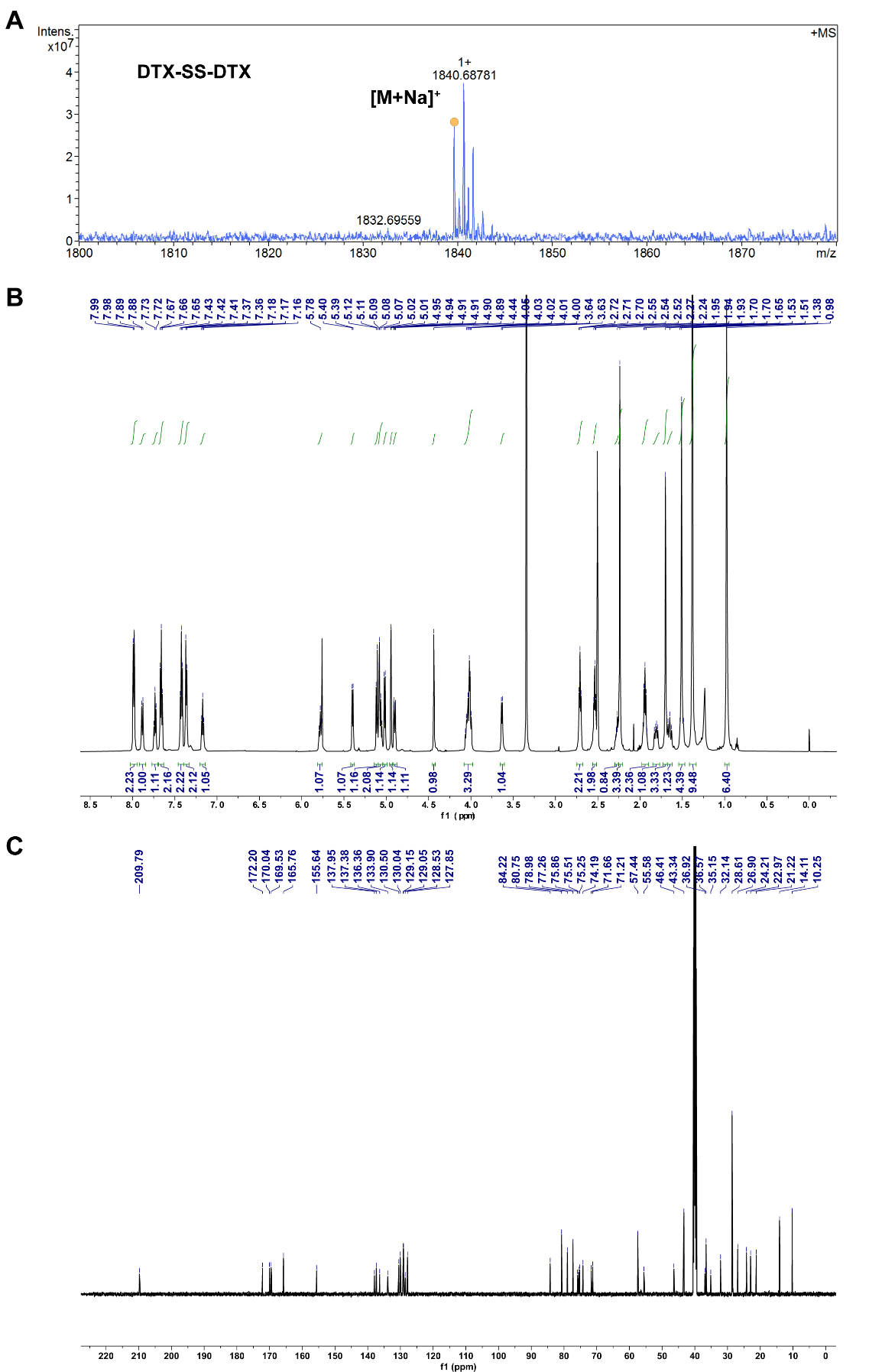


Figure S2. Structure confirmation of dimeric prodrug of docetaxel (DTX-SS-DTX). **(A)** Mass spectrum of DTX-SS-DTX. **(B)** ^1^H NMR spectrum of DTX-SS-DTX. (600 MHz, DMSO-d_6_) δ 7.99 (d, J = 7.1 Hz, 2H, H-23, H-27), 7.88 (d, J = 9.3 Hz, 1H, 3’-NH), 7.73 (t, J = 7.4 Hz, 1H, H-25), 7.66 (t, J = 7.6 Hz, 2H, H-24, H-26), 7.42 (t, J = 7.6 Hz, 2H, H-11’, H-13’), 7.36 (d, J = 7.6 Hz, 2H, H-10’, H-14’), 7.17 (t, J = 7.4 Hz, 1H, H-12’), 5.78 (t, J = 9.6 Hz, 1H, H-13), 5.40 (d, J = 7.2 Hz, 1H, H-2), 5.11 (d, J = 7.9 Hz, 1H, H-2’), 5.10-5.04 (m, 2H, H-3’, 10-OH), 5.02 (d, J = 7.2 Hz, 1H, H-3’), 4.94 (d, J = 2.4 Hz, 1H, H-5), 4.90 (dd, J = 9.5, 2.3 Hz, 1H, H-7), 4.44 (s, 1H, H-10), 4.08-3.98 (m, 3H, H-20α, H-20β, 7-OH), 3.63 (d, J = 7.1 Hz, 1H, H-3), 2.71 (t, J = 7.1 Hz, 2H, H-1’’), 2.54 (t, J = 7.2 Hz, 2H, H-3’’), 2.28 (dd, J = 9.0, 6.0 Hz, 1H, H-6α), 2.24 (s, 3H, H-29), 1.94 (p, J = 6.9 Hz, 2H, H-2’’), 1.81 (dd, J = 15.3, 9.3 Hz, 1H, H-6β), 1.70 (s, 3H, H-19), 1.65 (t, J = 12.4 Hz, 1H, H-14α), 1.52 (m, 1H, H-14β), 1.51 (s, 3H, H-19), 1.38 (s, 9H, H-6’, H-7’, H-8’), 0.98 (s, 6H, H-16, H-17). **(C)** ^13^C NMR spectrum of DTX-SS-DTX. (151 MHz, DMSO-d6) δ 209.79 (C-9), 172.20 (C-1’), 170.04 (C-28), 169.53 (C-4’’), 165.76 (C-21), 155.64 (C-4’), 137.95 (C-12), 137.38 (C-11), 136.36 (C-9’), 133.90 (C-25), 130.50 (C-22), 130.04 (C-23, C-27), 129.15 (C-24, C-26), 129.05 (C-11’, C-13’), 128.53 (C-12’), 127.85 (C-10’, C-14’), 84.22 (C-2’), 80.75 (C-5), 78.98 (C-1), 77.26 (C-5’), 75.86 (C-4), 75.51 (C-2), 75.25 (C-20), 74.19 (C-10), 71.66 (C-7), 71.21 (C-13), 57.44 (C-8), 55.58 (C-3’), 46.41 (C-3), 43.34 (C-15), 36.92 (C-1’’), 36.57 (C-14), 35.15 (C-6), 32.14 (C-3’’), 28.61 (C-6’, C-7’, C-8’), 26.90 (C-2’’), 24.21 (C-16), 22.97 (C-17), 21.22 (C-29), 14.11 (C-18), 10.25 (C-19).


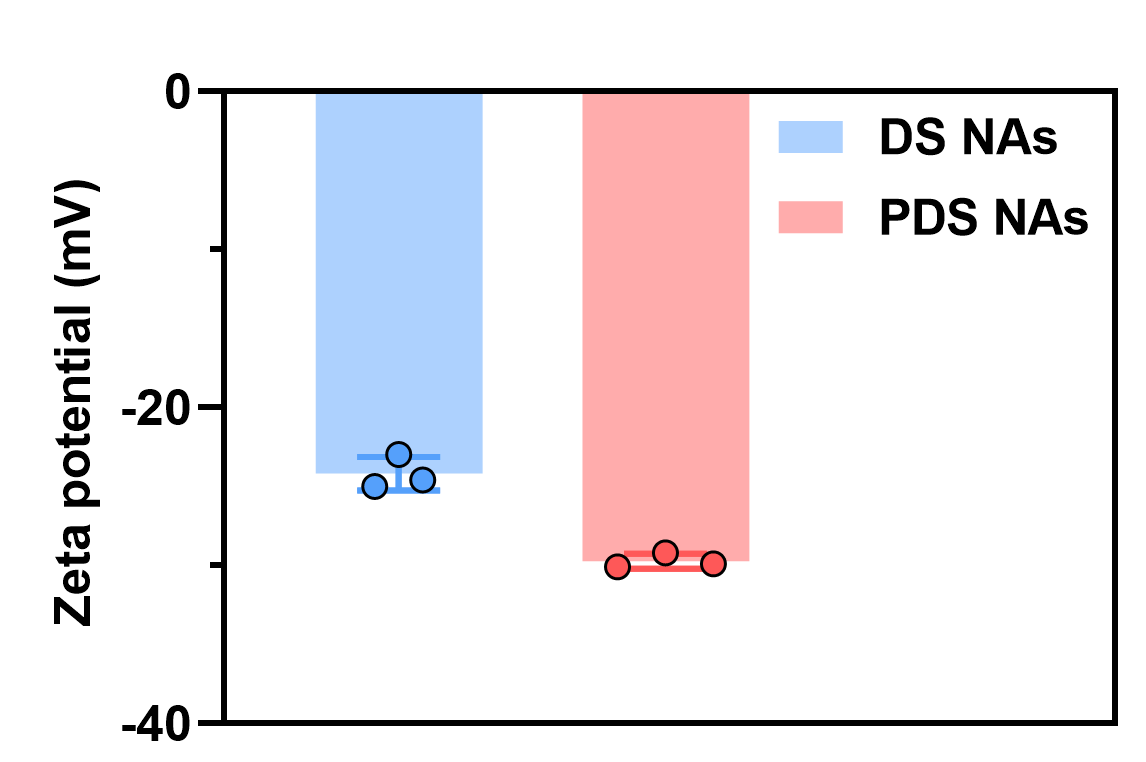


Figure S3. *Zeta* potentials of DS NAs and PDS NAs (n = 3). Data are presented as mean ± SD.


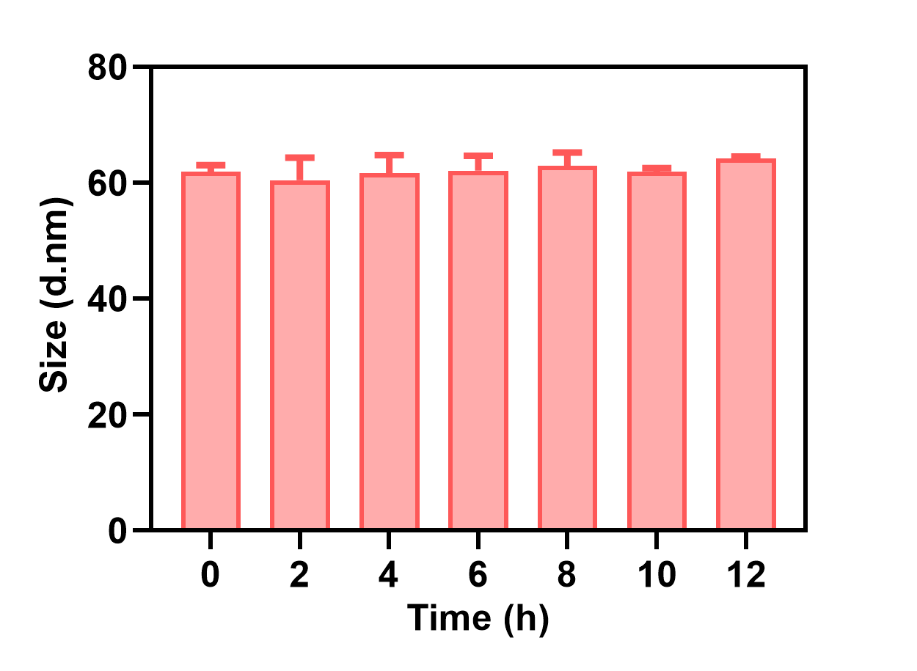


Figure S4. Stability of PDS NAs incubated in PBS (pH 7.4) containing 10% FBS (n = 3). Data are presented as mean ± SD.


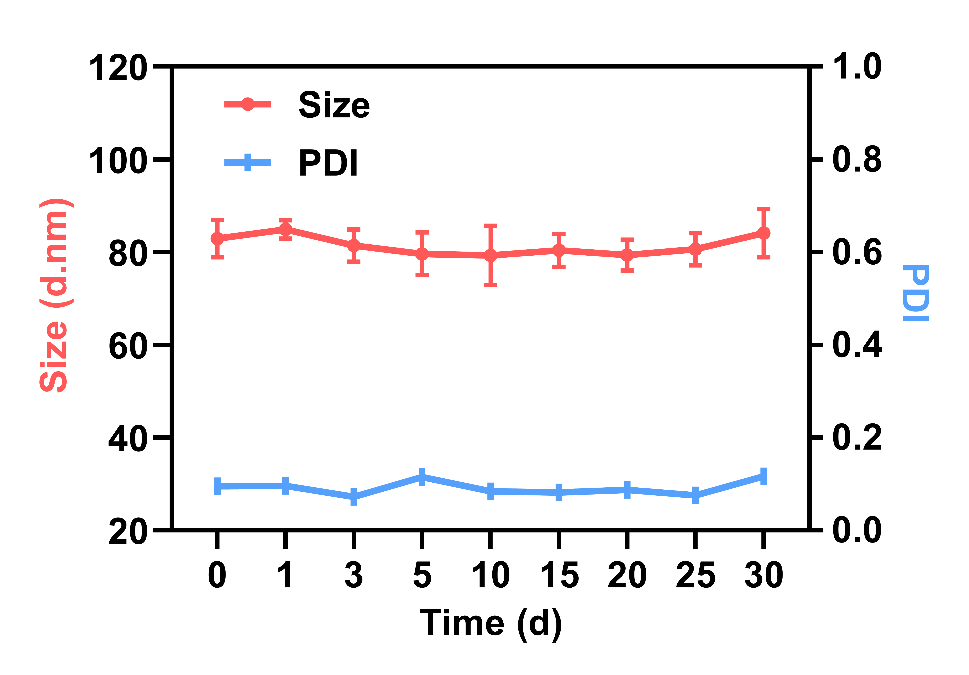


Figure S5. Long-term storage stability of PDS NAs after being stored at 4℃ for 30 days (n = 3). Data are presented as mean ± SD.


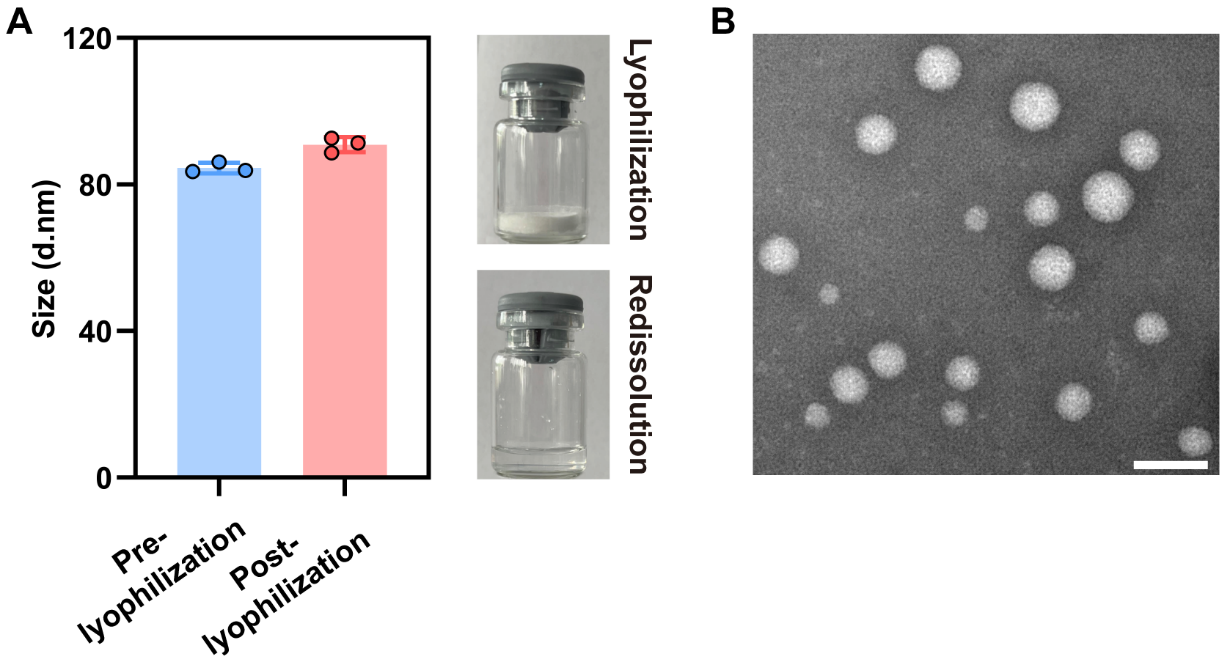


Figure S6. **(A)** Particle size and representative images of PDS NAs before and after lyophilization (n = 3). Data are presented as mean ± SD. **(B)** TEM image of PDS NAs after lyophilization. Scale bar represents 100 nm.


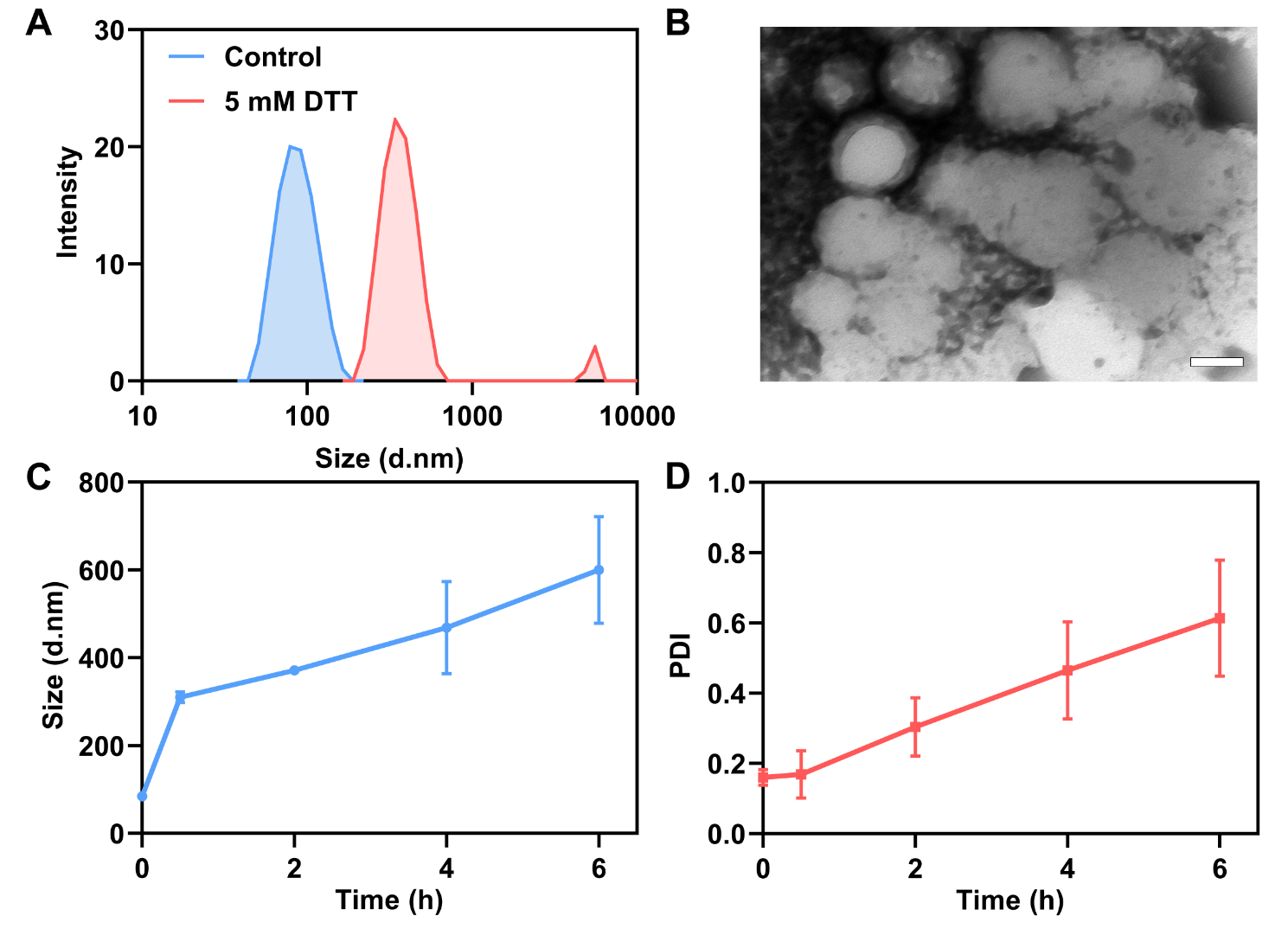
Figure S7. **(A)** Intensity size distribution profiles and **(B)** TEM image of PDS NAs in the presence of 5 mM DTT. Scale bar represents 100 nm. **(C)** Changes in particle size and **(D)** particle distribution index (PDI) of PDS NAs at different time points after incubation with 5 mM DTT (n = 3). Data are presented as mean ± SD.


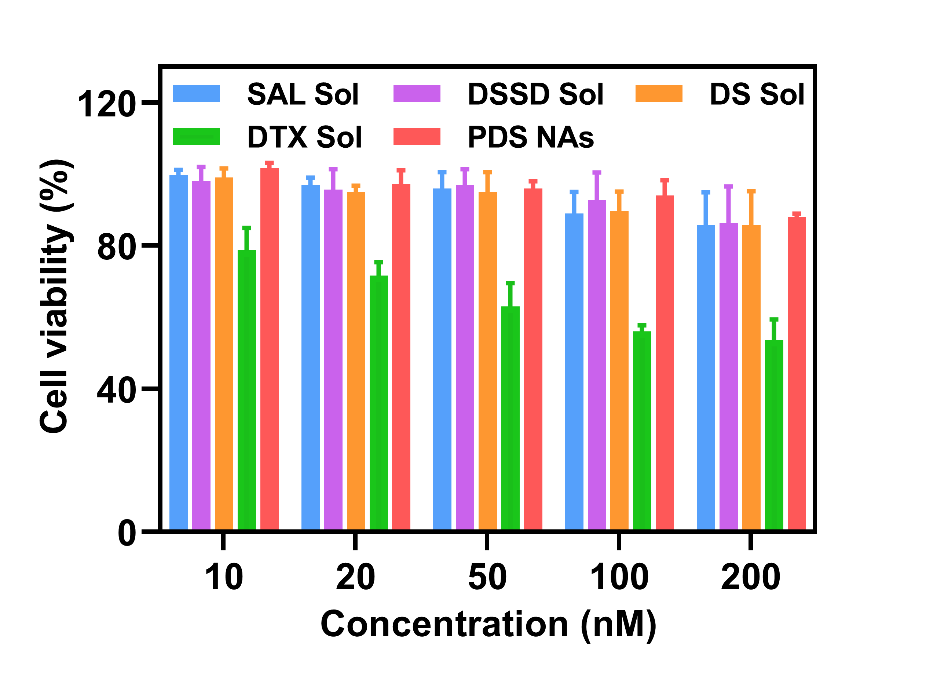


Figure S8. Cell viability of L02 cells under various formulations (n = 3). Data are presented as mean ± SD.


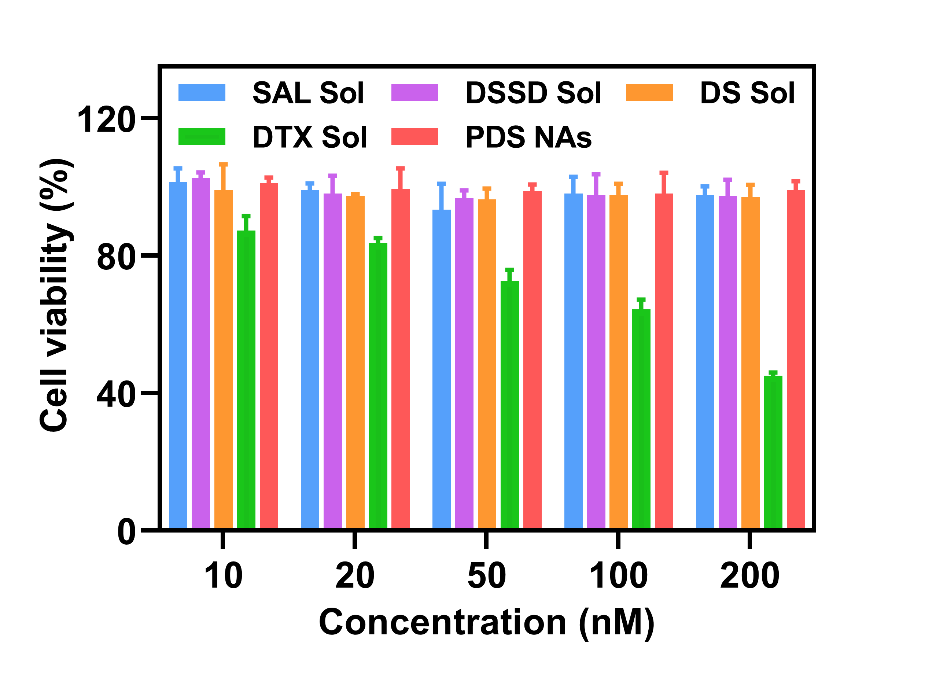


**Figure S9.** *In vitro* cytotoxicity of various formulations against 3T3 normal cells (n = 3). Data are presented as mean ± SD.


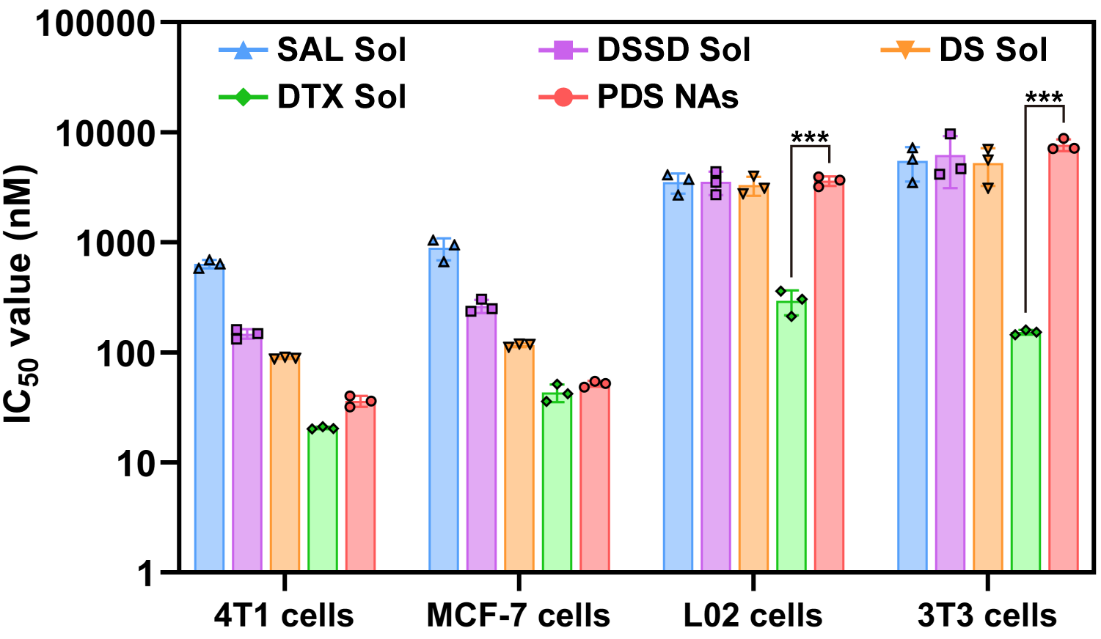


Figure S10. IC_50_ values of various formulations against 4T1 cancer cells, MCF-7 cancer cells, L02 normal cells, and 3T3 normal cells. Data are presented as mean ± SD (n = 3). p values: ***p < 0.001.


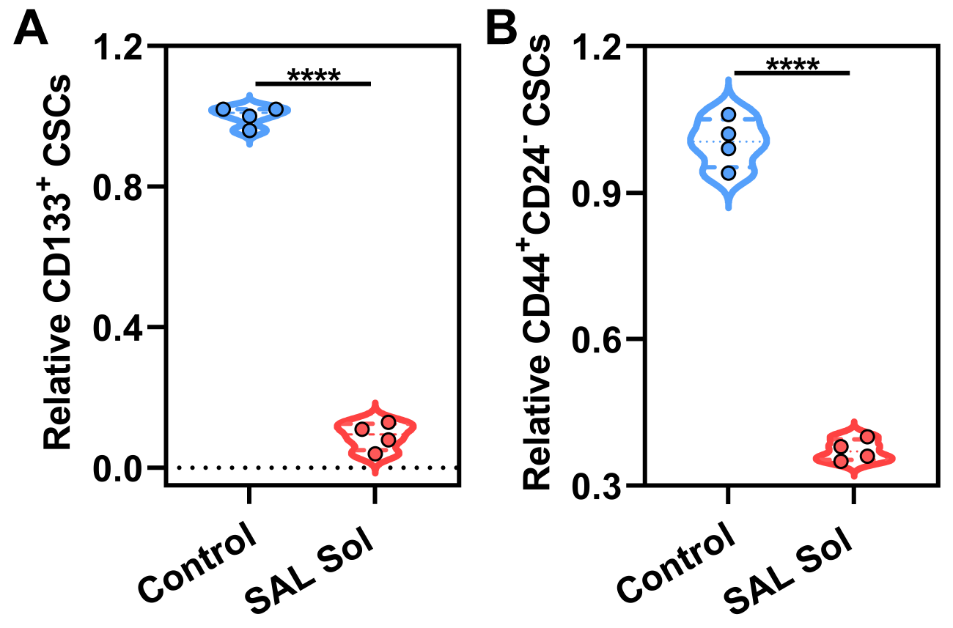


Figure S11. The proportion of CSCs in 4T1 cancer cells after co-incubation with SAL for 48 h. **(A)** The quantification of CD133^+^ CSCs in 4T1 cancer cells following treatment with SAL (n = 4). **(B)** The quantification of CD44^+^CD24^-^ CSCs in 4T1 cancer cells following treatment with SAL (n = 4). p values: ****p < 0.0001.


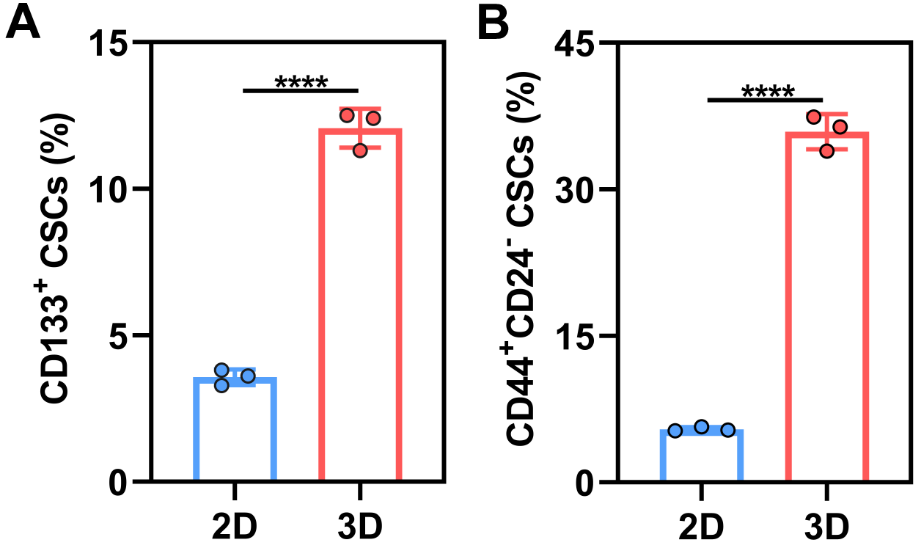


Figure S12. **(A)** The percentage of CD133^+^ CSCs in 4T1 cancer cells and 3D mammospheres (n = 3). **(B)** The percentage of CD44^+^CD24^-^ CSCs in 4T1 cancer cells and 3D mammospheres (n = 3). Data are presented as mean ± SD. p values: ****p < 0.0001.


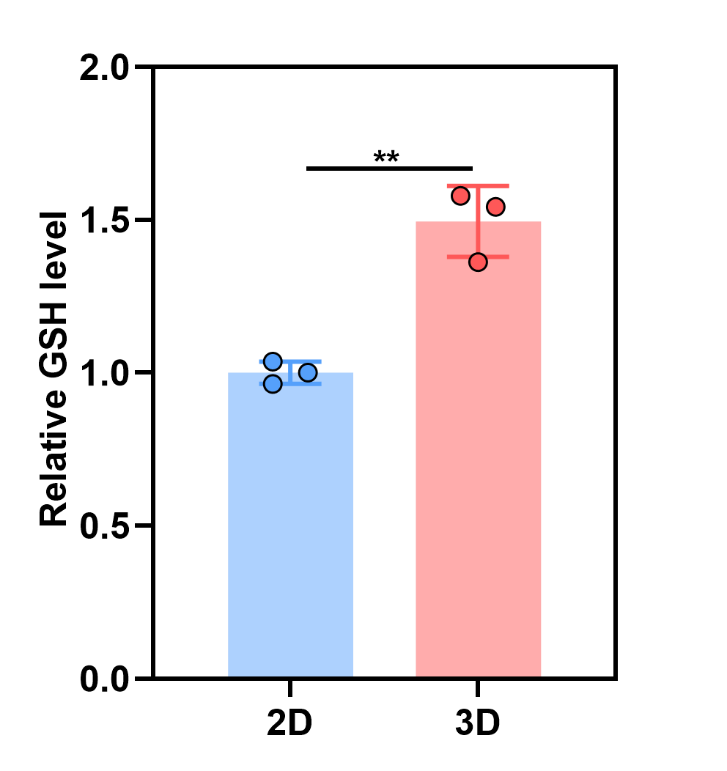


Figure S13. Relative GSH levels in 4T1 cancer cells and CSCs (n = 3). Data are presented as mean ± SD. p values: **p < 0.01.


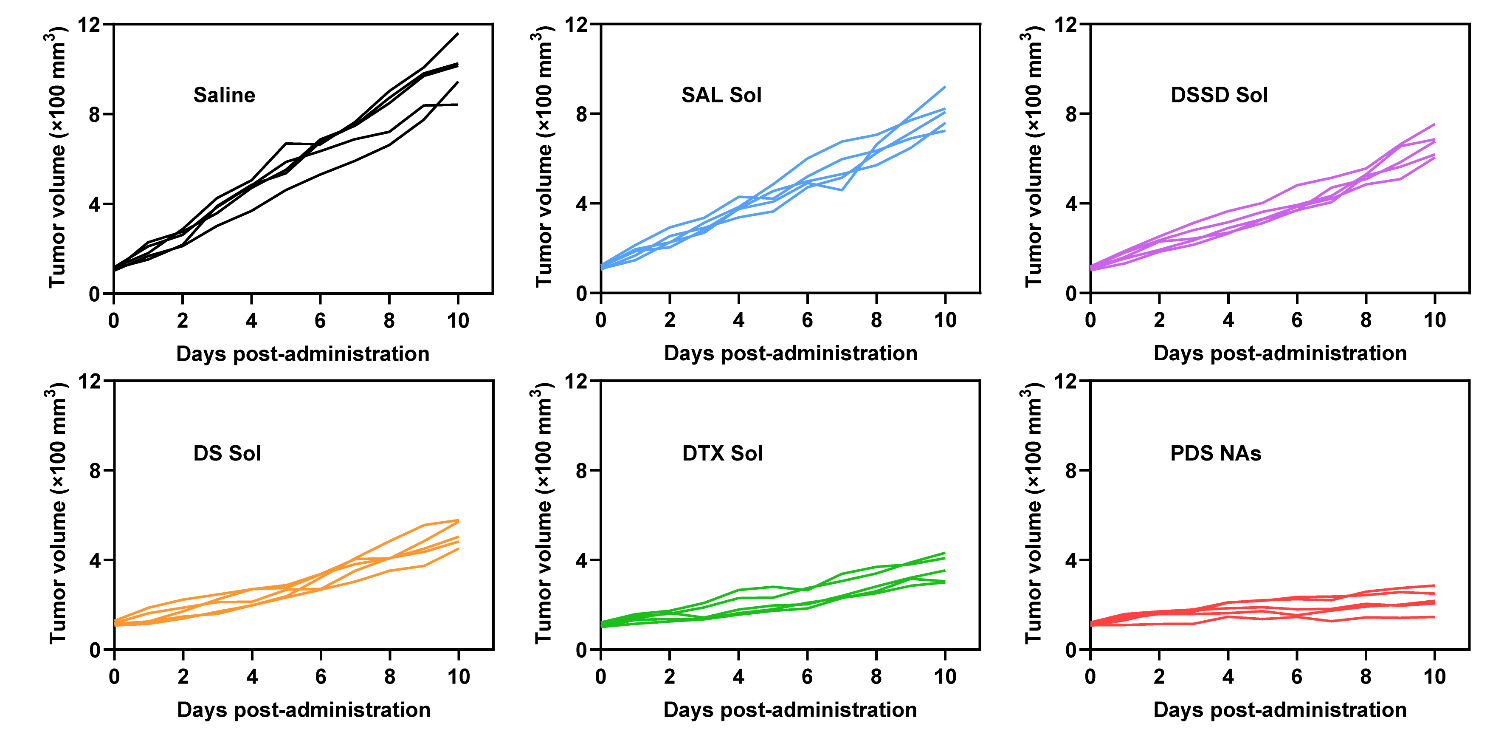


Figure S14. Tumor growth curves of each 4T1 xenograft tumor-bearing mouse after different treatments (n = 5).

**
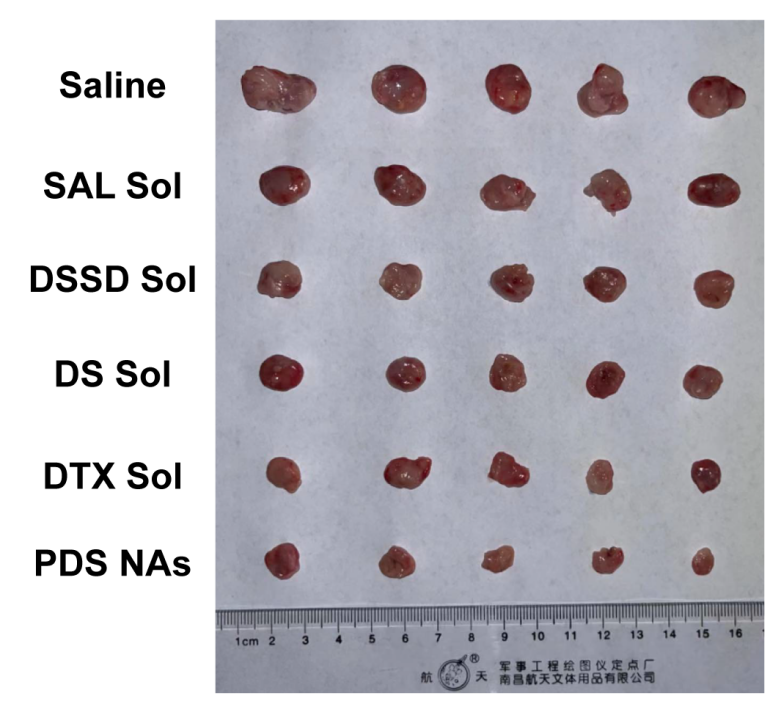
**

Figure S15. Image of tumors following the final treatment (n = 5).


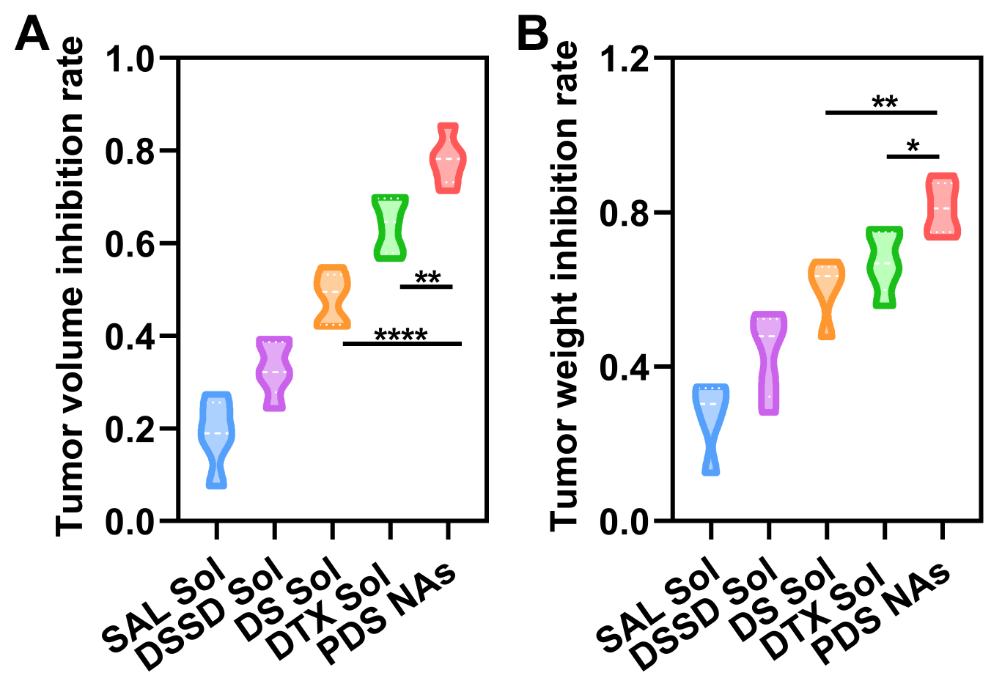


**Figure S16.** (A) Tumor volume and (B) weight inhibition rate of different treatment groups (n = 5).

p values: *p < 0.05, **p < 0.01, ****p < 0.0001


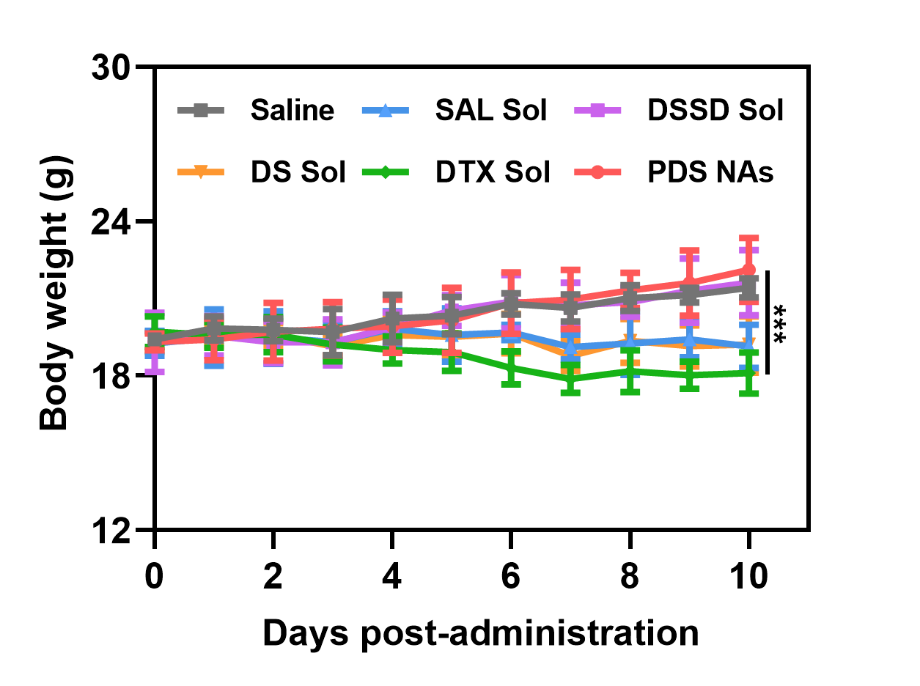


**Figure S17.** Changes in body weight of 4T1 xenograft tumor-bearing mice during treatment periods (n = 5). Data are presented as mean ± SD. p values: ***p < 0.001.


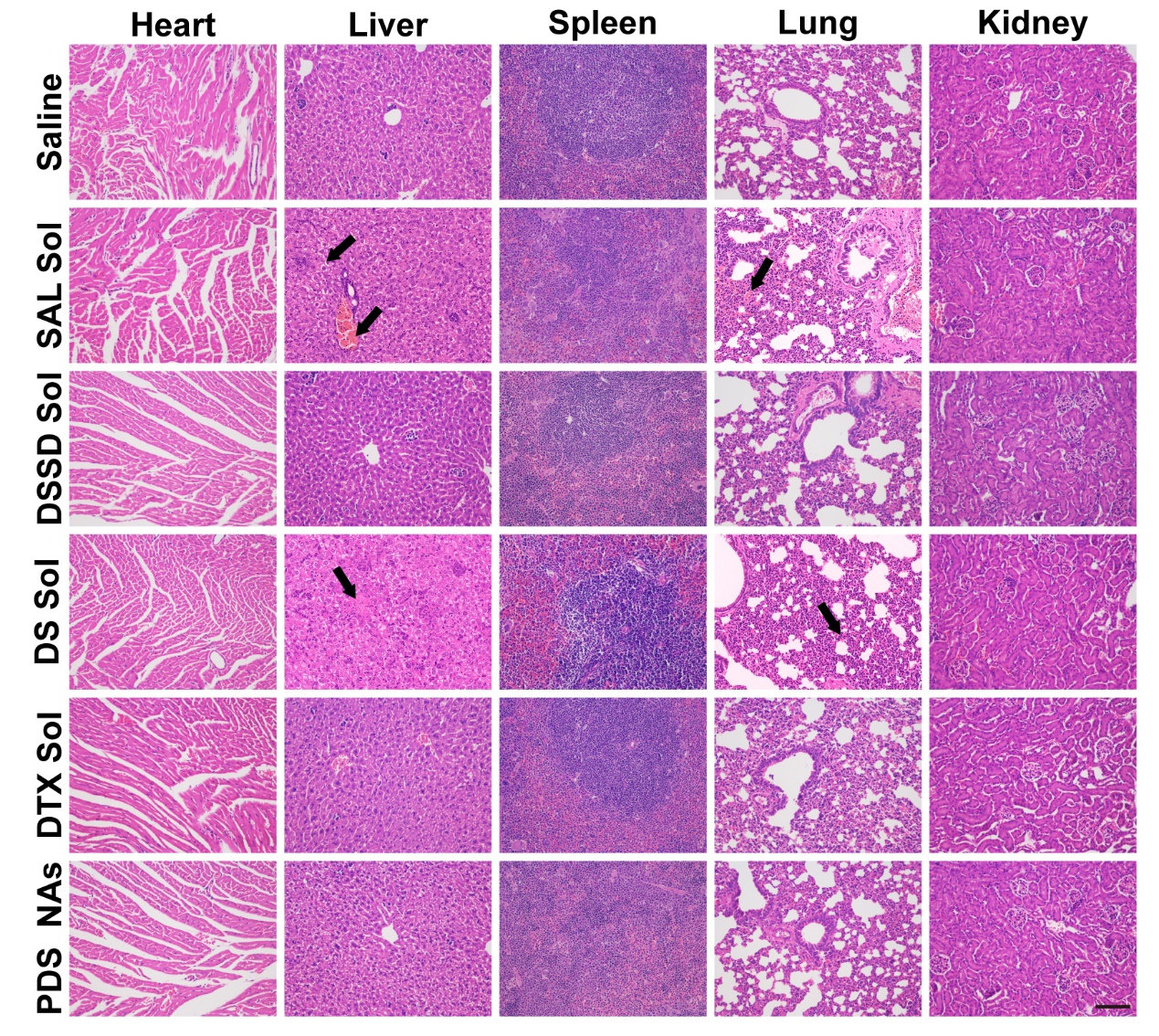


Figure S18. H&E staining images of heart, liver, spleen, lung, and kidney of 4T1 xenograft tumor-bearing mice after different treatments. Scale bar represents 100 μm.


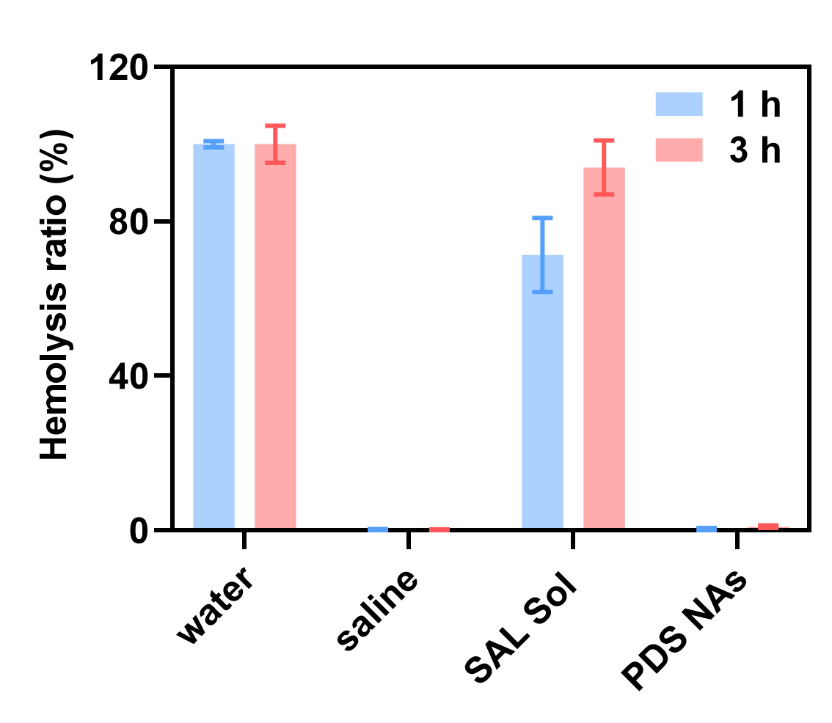


Figure S19. The hemolysis ratio of red blood cells after treatment with SAL Sol or PDS NAs for 1 h and 3 h (n = 3). Saline and water were used as the negative and positive controls, respectively. Data are presented as mean ± SD.


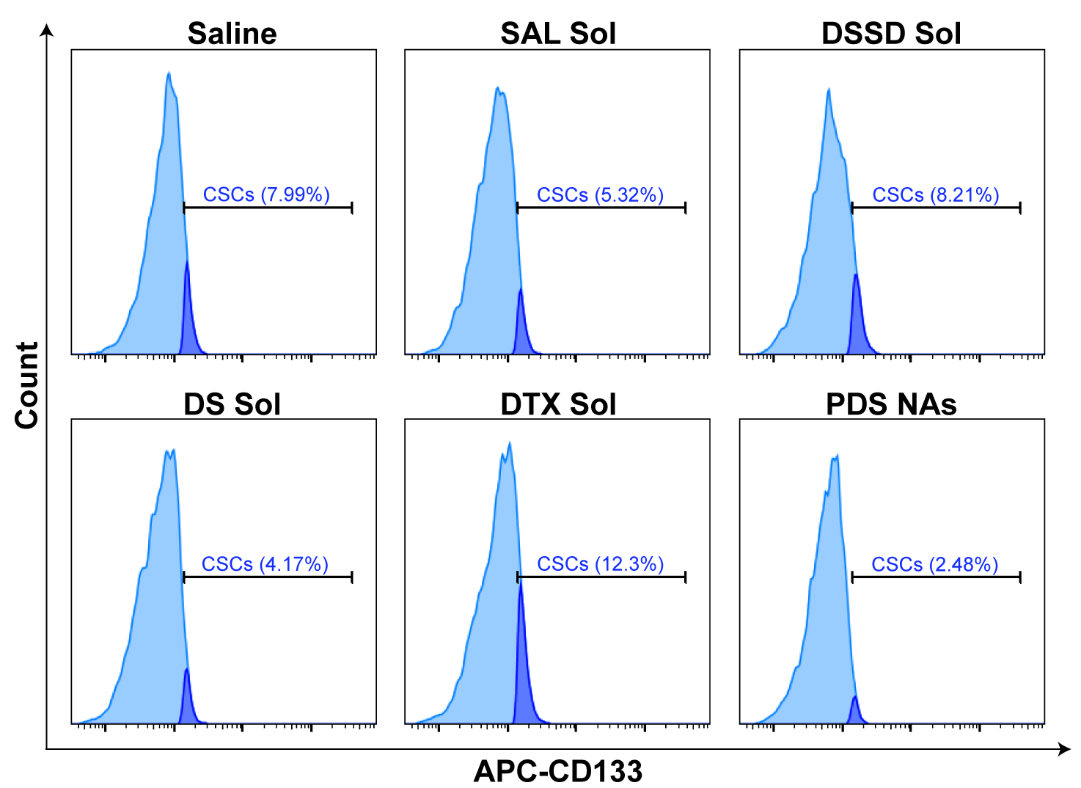


Figure S20. The proportion of CSCs characterized CD133^+^ detected by flow cytometry.


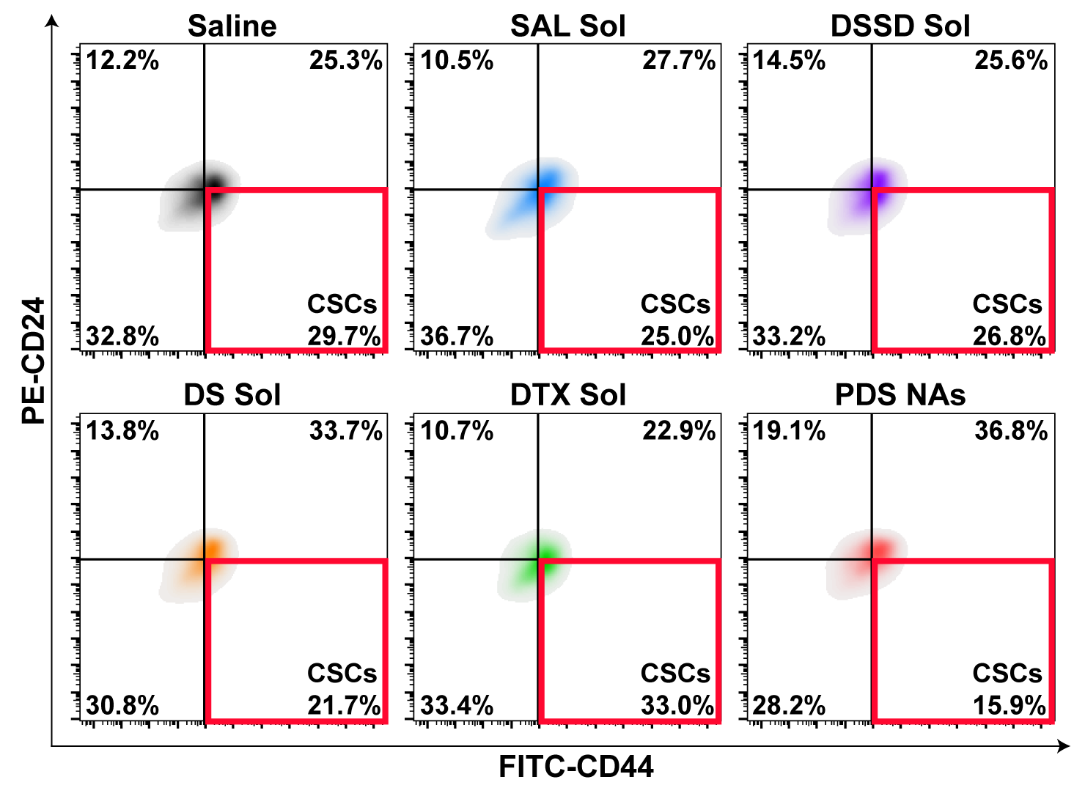


Figure S21. The percentage of CD44^+^CD24^-^ CSCs in tumor tissues detected by flow cytometry.


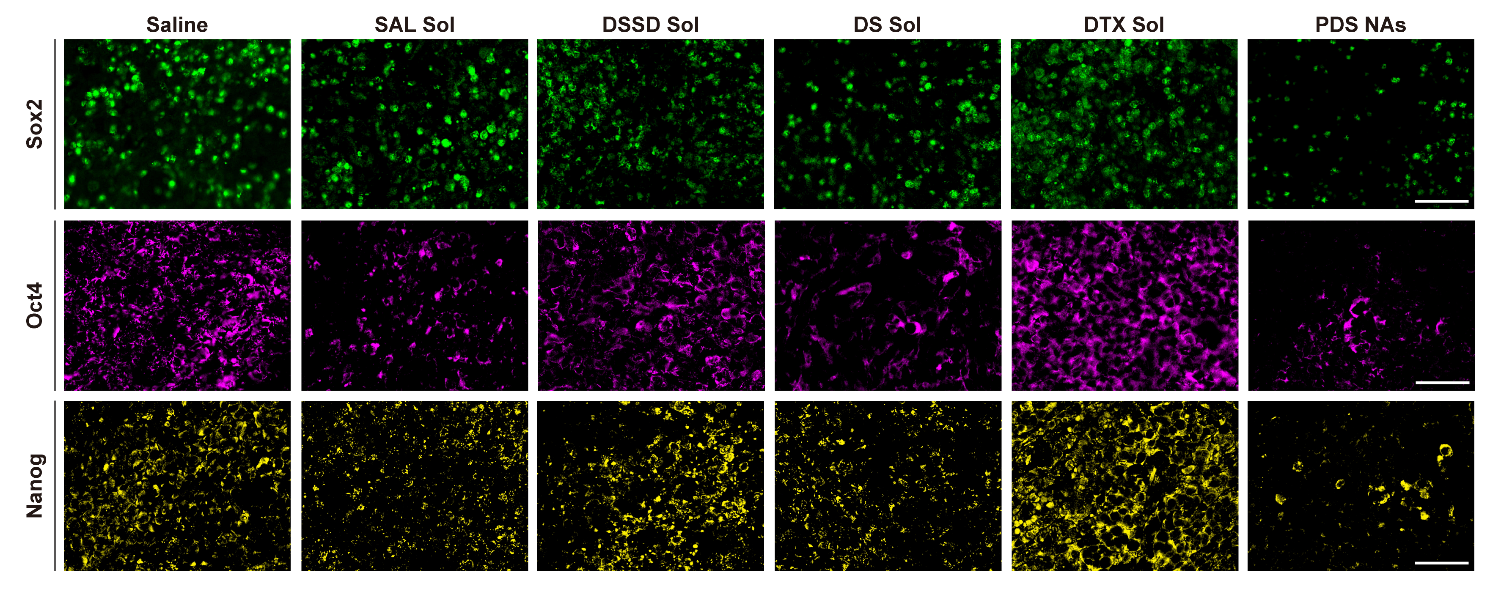


Figure S22. Immunofluorescence-stained images of the tumor sections with three CSC pluripotency factors (Sox2, Oct4 and Nanog). Scale bar represents 100 μm.


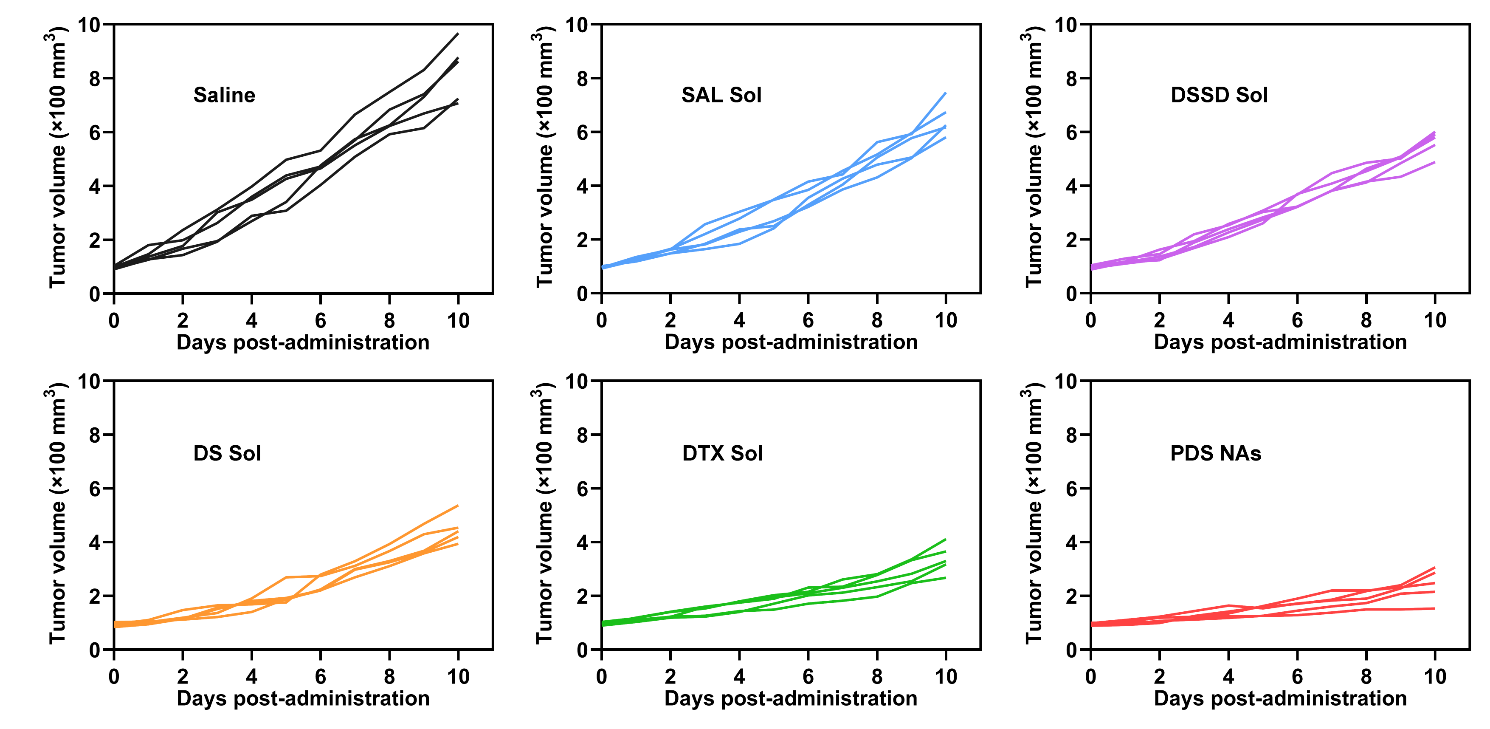
Figure S23. Tumor growth curves of each 4T1 orthotopic tumor-bearing mouse after different treatments (n = 5).


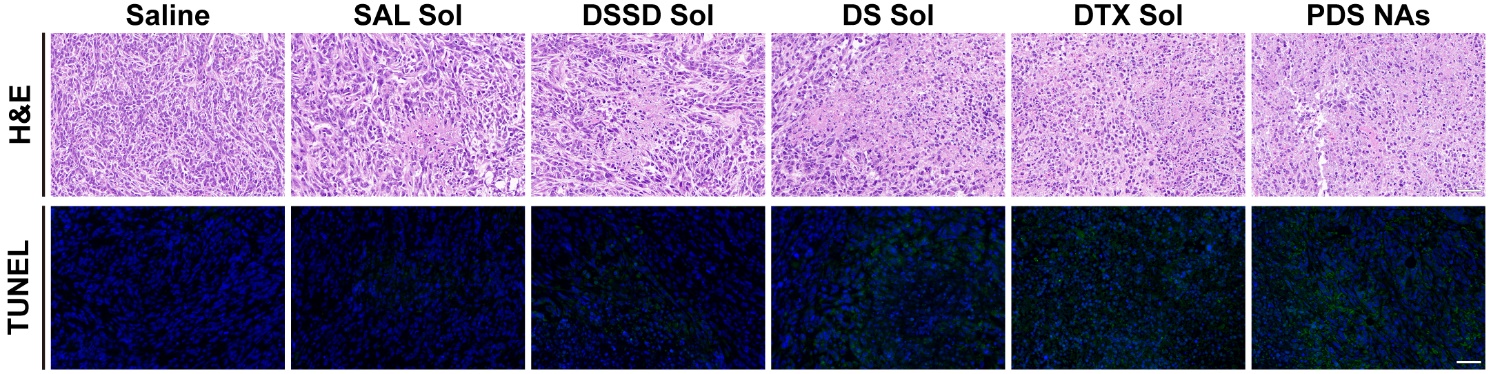
Figure S24. H&E and TUNEL staining images of tumor slices from 4T1 orthotopic tumor-bearing mice after different treatments. Scale bar represents 50 μm.


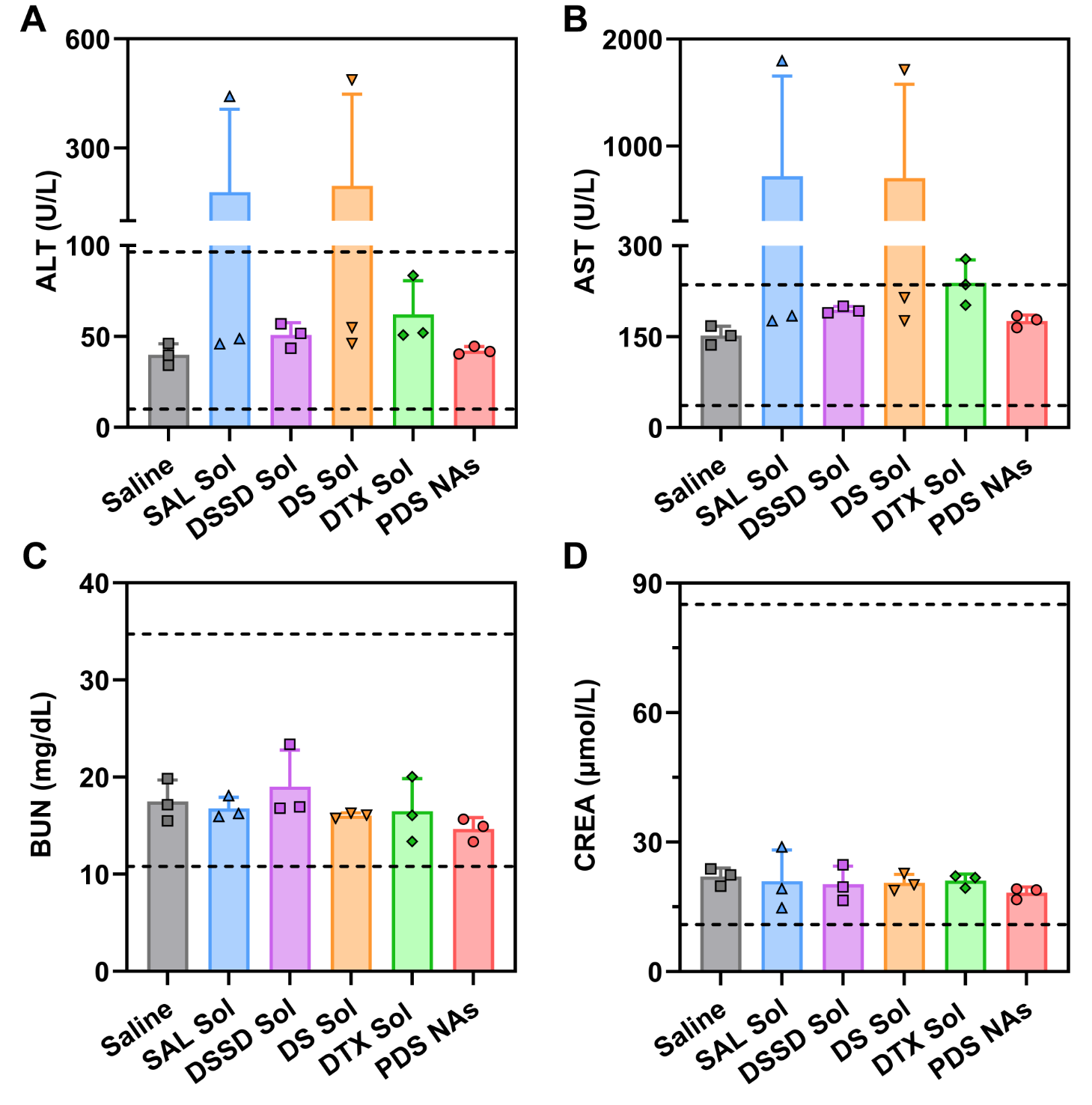


**Figure S25.** Hepatic and renal function indicators of 4T1 orthotopic tumor-bearing mice after different treatments (n = 3). Data are presented as mean ± SD.


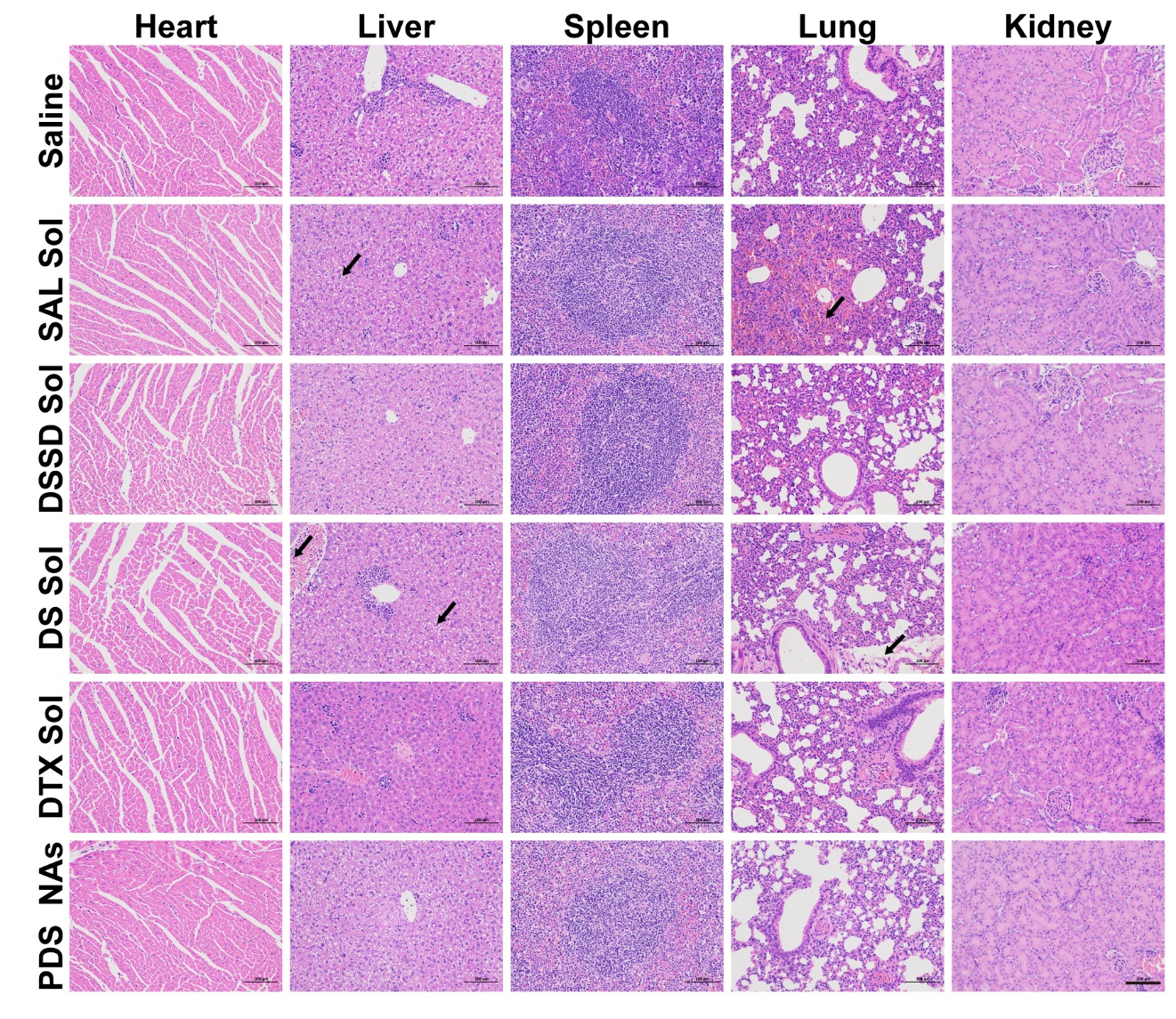


**Figure S26.** H&E staining images of heart, liver, spleen, lung, and kidney from 4T1 orthotopic tumor-bearing mice after different treatments. Scale bar represents 100 μm.


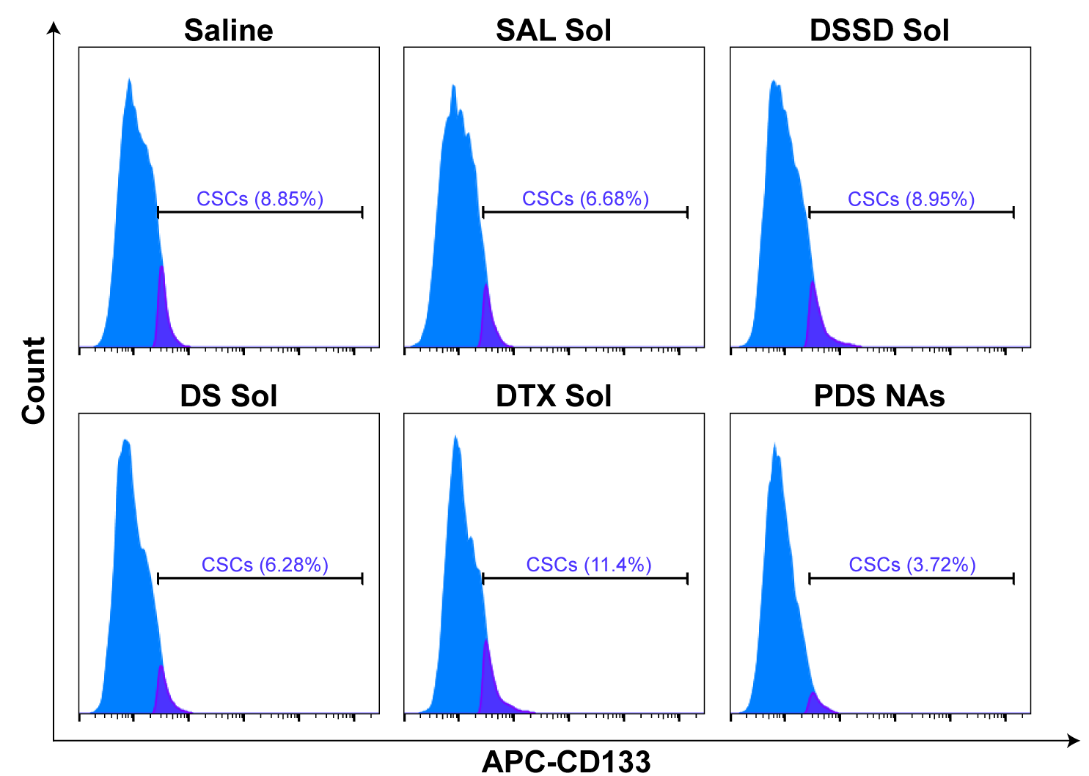


**Figure S27.** The proportion of CD133^+^ CSCs in tumor tissues after treatment with various formulations.


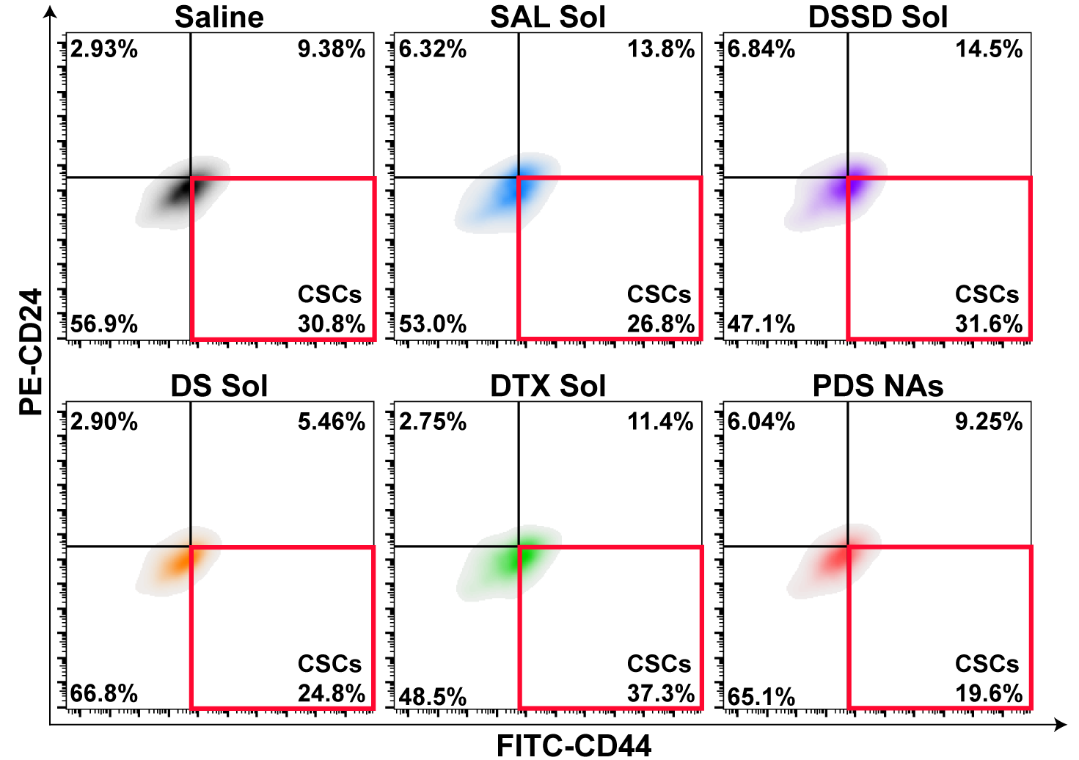


Figure S28. The percentage of CD44^+^CD24^-^ CSCs in tumor tissues following treatment with different formulations.

Table S1. Characterization of the non-PEGylated nanoassemblies (n = 3).

| Formulations  DSSD/SAL (mol/mol) | Size (nm) | PDI |
| --- | --- | --- |
| DSSD | NA | NA |
| SAL | NA | NA |
| DSSD/SAL = 5:1 | 289.9 ± 7.508 | 0.243 ± 0.018 |
| DSSD/SAL = 4:1 | 333.4 ± 10.55 | 0.293 ± 0.024 |
| DSSD/SAL = 3:1 | 348.8 ± 16.64 | 0.208 ± 0.077 |
| DSSD/SAL = 2:1 | 207.2 ± 4.431 | 0.073 ± 0.083 |
| DSSD/SAL = 1:1 | 120.9 ± 2.052 | 0.099 ± 0.052 |
| DSSD/SAL = 1:2 | 164.3 ± 1.365 | 0.077 ± 0.054 |
| DSSD/SAL = 1:3 | 133.6 ± 2.836 | 0.102 ± 0.072 |
| DSSD/SAL = 1:4 | 134.6 ± 1.168 | 0.106 ± 0.018 |
| DSSD/SAL = 1:5 | 121.5 ± 1.815 | 0.152 ± 0.049 |

NA: Not available.

Table S2. Screening the synergistic dose ratio of DSSD and SAL through cytotoxicity assessment.

| Formulations  DSSD/SAL (mol/mol) | Cooperativity index (CI) |
| --- | --- |
| 5:1  4:1  3:1  2:1  1:1  1:2  1:3  1:4  1:5 | 0.570 |
|  | 0.440 |
|  | 0.422 |
|  | 0.341 |
|  | 0.294 |
|  | 0.378 |
|  | 0.384 |
|  | 0.396 |
|  | 0.496 |

Table S3. Characterization of the PEGylated nanoassemblies (DSSD/SAL = 1:1) with different proportions of DSPE-PEG_2K_ (n = 3).

| Formulations | Size (nm) | PDI | *Zeta* potentials (mV) |
| --- | --- | --- | --- |
| 10% | 99.32 ± 0.835 | 0.054 ± 0.047 | -24.8 ± 0.84 |
| 20% | 81.66 ± 1.153 | 0.095 ± 0.051 | -30.1 ± 1.20 |
| 30% | 99.05 ± 0.745 | 0.108 ± 0.044 | -32.1 ± 0.96 |

Table S4. Drug loading capacity of non-PEGylated nanoassemblies (DS NAs) and PEGylated nanoassemblies (PDS NAs).

| Nanoassemblies | Drug loading of DTX | Drug loading of SAL |
| --- | --- | --- |
| DS NAs | 63% | 29% |
| PDS NAs | 50% | 23% |

Table S5. Pharmacokinetic parameters of DiR Sol and DiR/PDS NAs (n = 6).

| Formulations | AUC_0-24 h_ (μg/mL*h)) | C_max_ (μg/mL) |
| --- | --- | --- |
| DiR Sol | 1.172 ± 0.389 | 0.275 ± 0.157 |
| DiR/PDS NAs | 54.897 ± 8.747 | 12.541 ± 1.837 |
